# An insight into structural plasticity and conformational transitions of transcriptional co-activator Sus1

**DOI:** 10.1371/journal.pone.0229216

**Published:** 2020-03-05

**Authors:** Akhilendra Pratap Bharati, Mohd Kashif, Sumit Kumar Chaturvedi, Rizwan Hasan Khan, Abrar Ahmad

**Affiliations:** 1 ICAR–National Bureau of Agriculturally Important Microorganisms, Mau, Uttar Pradesh, India; 2 Center for Plant Molecular Biology Division, CSIR-NBRI, Lucknow, India; 3 Interdisciplinary Biotechnology Unit, Aligarh Muslim University, Aligarh, India; 4 Department of Biochemistry, Faculty of Science, King Abdulaziz University, Jeddah, Kingdom of Saudi Arabia; University of Hyderabad School of Life Sciences, INDIA

## Abstract

RNA biogenesis and mRNA transport are an intricate process for every eukaryotic cell. SAGA, a transcriptional coactivator and TREX-2 are the two major complexes participate in this process. Sus1 is a transcription export factor and part of both the SAGA and the TREX-2 complex. The competitive exchange of Sus1 molecule between SAGA and TREX-2 complex modulates their function which is credited to structural plasticity of Sus1. Here, we portray the biophysical characterization of Sus1 from *S*. *cerevisiae*. The recombinant Sus1 is a α-helical structure which is stable at various pH conditions. We reported the α-helix to β-sheet transition at the low pH as well as at high pH. Sus1 showed 50% reduction in the fluorescence intensity at pH-2 as compared to native protein. The fluorescence studies demonstrated the unfolding of tertiary structure of the protein with variation in pH as compared to neutral pH. The same results were obtained in the ANS binding and acrylamide quenching studies. Similarly, the secondary structure of the Sus1 was found to be stable till 55% alcohol concentration while tertiary structure was stable up to 20% alcohol concentration. Further increase in the alcohol concentration destabilizes the secondary as well as tertiary structure. The 300 mM concentration of ammonium sulfate also stabilizes the secondary structure of the protein. The structural characterization of this protein is expected to unfold the process of the transportation of the mRNA with cooperation of different proteins.

## Introduction

A major part of mRNA biogenesis occurs co-transcriptionally and it is associated with the various steps of transcription like chromatin modifications, transcriptional elongation, splicing and mRNA export [1,2]. Sus1 is a small 11 kDa protein which is the part of the two mega complex like TREX2 and SAGA. This is highly conserved across the species and has a role in transcription as well as in mRNA export [3,4]. It plays a major role in the mRNA export by anchoring actively transcribing genes from the nuclear periphery through gene gating. Sus1 is involved in the mRNA export coupled transcription activation through the association of SAGA and TREX2 complexes [5]. Sus1 is involved at the different stage of gene expression and mRNA biogenesis through the interaction with the carboxy terminal domain (CTD) of RNA polymerase II [6]. SAGA complex and histone acetyl transferase (HAT) in addition with the CTD phosphorylation regulate transcription elongation in the yeast [7]. Sus1 as part of the SAGA complex, also found to be involved in chromatin modification, where it plays a major role in histone H2B deubiquitinating (DUB) activity together with Ubp8, Sgf11 and Sgf73 [7–9].

In addition, during the transcription of many genes the Sus1 is recruited to the coding region of the genes through the association with the CTD of RNA polymerase II,Yra1 and Max67 [10]. At the nuclear pore the Sus1 forms a complex with the Sac3, Thp1, Cdc31 and Sem1 which is termed as TREX2 complex. This complex interacts with the component of mRNA export machinery (Mex6, Mtr2 and Sub2) in the nucleoplasm and with the nucleoporin (Nup1) in the nuclear basket [11–13]. Through these complexes it is involved in the anchoring the actively transcribing genes at the nuclear periphery and transport through the gene gating [13,14]. In addition to the mRNA biogenesis, it is involved in the maintenance of the genome integrity by preventing RNA mediated genome instability [15].

Most of the yeast genes don’t contain introns, but Sus1 have two introns. Most yeast introns display conserved splicing signals. These introns have been studied widely and found to be conserved throughout the evolution, their deletion leads to the defect is the mRNA export, and is detrimental for the cell growth [16]. Sus1 has also a role in the DNA damage response (DDR) induced by the methyl methane sulfonate as well as in the replication arrest induced by hydroxyurea. Crystallography has provided important information regarding the structural changes in the Sus1 when interacts with other proteins of SAGA and TREX complexes [17]. However, proteins are not static, and some conformational species may not be represented in the different crystalline forms.

Here, we have described cloning, expression, physicochemical characterization of Sus1 protein from *S*. *cerevisiae*. Sus1 protein contains single tryptophan residue which makes it an interesting protein for fluorescence and CD studies. We have performed the folding studies of Sus1 protein at different pH condition which showed the stability in the secondary structure at lower as well as higher pH. It also showed the transition between the α-helix and β-sheets at low and high pH. The intrinsic fluorescence studies along with the ANS fluorescence and acrylamide quenching showed the unfolding of the tertiary structure in varying pH conditions as compared to pH-7. The different concentration of the alcohol and ammonium sulfate (denaturants) showed the stabilization of the secondary as well as tertiary structure till a minimal concentration.

## Material and method

### Cloning, expression and purification of Sus1

*SUS*1 gene was PCR amplified from the c-DNA of *Saccharomyces cerevisiae* using specific primers (forward- 5' CTAGCTAGCATGGTATTGGCAATGGAAAGTAGAGTGGCA-3' and reverse-5'-ATAA GAATGCGGCCGCGTCAGACCAATCATCCTCATCTA-3') and cloned into pET-21d (+) expression vector (Novagen) between *Nhe*I and *Xho*I restriction site. The *E*. *coli* (BL21-DE3) was transformed with the expression construct. The protein was expressed and purified using Ni-NTA column as described somewhere [18, 19]. Further purification was done using Superdex^TM^ 75, 10/300GL column on AKTA FPLC (GE Healthcare) pre-calibrated with standard molecular weight markers. Finally, the protein was dialysed with 20 mM Tris-HCl buffer at pH 7.0, 100 mM NaCl and 10% Glycerol.

### Physico-chemical characterization of Sus1

To check the effect of pH on the protein structure the buffer of different pH was prepared. For pH-1 KCl-HCl buffer, for pH 2.0–3.0 Glycine-HCl buffer and similarly for pH 3.0–5.0, pH-6.0, pH 7.0–8.0, sodium acetate buffer, sodium phosphate buffer and tris-HCl buffer were used respectively. A stock solution of 1.5 mg/ml protein was prepared in 20 mM Tris-HCl buffer at pH 7.0, 100 mM NaCl and 10% Glycerol and was properly dialyzed with the desired buffer. Protein concentration was determined by Bradford kit. Mettler Toledo pH meter (model seven easy S 20) was used for the pH measurements. Protein was incubated for 12 hours at desired buffer before spectroscopic measurements were recorded.

### Circular dichroism measurements

All the circular dichroism (CD) spectra were taken using the JASCO J-815 spectrophotometer calibrated with the d-10-camphorsulphonic acid. This instrument was associated with Jasco peltier type temperature controller (PTC-424S/15). Far-UV CD spectra were collected in a cell of 1 mm with the 100 nm/min scan speed in the response time of 1 sec. Each spectrum was the average scan of the three scans and spectra were smoothed by the Savitzky–Golay method with 25 convolution width. Each spectrum was measured at the 0.15 mg/ml protein concentration. The CD result has been expressed in the mean residue ellipticity (MRE) in deg cm^2^ dmol^-1^ which is defined as:

$$MRE=\theta_{obs}\left(10\times n\times l\times Cp\right)$$

Here θ_obs_ stands for the CD in milli-degree, n stands for the number of amino acid residues, *l* stands for the path length of the cell, and Cp stands for the molar concentration of Sus1. Using the MRE values of 190–240 nm and molecular weight of the protein the % of α-helical content was calculated using the K2D3 web server [20, 21]

### Intrinsic and extrinsic fluorescence

All the fluorescence spectra were generated using ShimadzuRF-5301 spectrofluorometer. For the fluorescence study 0.15 mg/ml protein concentration was taken in 1 cm path length cuvette. Prior to performing the scan, samples were equilibrated with the desired concentration of organic solvent (ethanol) or salt (Sodium Sulphate) and incubated for 30 minutes at room temperature. The 290 nm of excitation wavelength was taken and the spectra were recorded between 300–450 nm, with 5 nm slit width for both the monochromators. ANS (8-Anilinonaphthalene-1-sulfonic acid) binding experiment was performed in the molar ration of 1:50 of protein and ANS respectively. The 50 fold molar excess of the ANS was incubated with the protein for 30 min at 25°C in the dark. The emission spectra were recorded in the range of 400–600 nm while the excitation wavelength was set to 380 nm [22,23].

### Acrylamide-quenching experiments

The acrylamide quenching experiment was performed at different concentration of the acrylamide (0.1–0.4 M). To achieve this concentration in the protein samples the stock solution of (2.5–1 M) acrylamide was first prepared and added to the protein solution. The only trp residue was excited using 290nm wavelength and the emission spectra were recorded between 300–400 nm wavelength. The spectra were recorded as described earlier in intrinsic and extrinsic fluorescence section. The data were plotted between the ratios of decrease in the fluorescence intensity versus acrylamide concentration and the relation between two were correlated using Stern-Volmer equation described below.

$$F_{o}/F=1+Ksv\;\left[Q\right]$$

$$Kq=Ksv/\tau_{0}$$

The F_o_ represents the initial fluorescence intensity (FI) without quencher while F represents the FI in the presence of quencher. Ksv is a constant known as quenching constant which can be calculated by the slope of the Stern-Volmer equation. The Q represents the concentration of the quencher. Kq represent the bimolecular rate constant of the quenching reaction. The τ_0_ represents the average integral fluorescence life time of the tryptophan, which is 4.31 *×* 10^-9^s [24].

## Results

### Purification of recombinant Sus1 and its native structure

**Fig 1:**
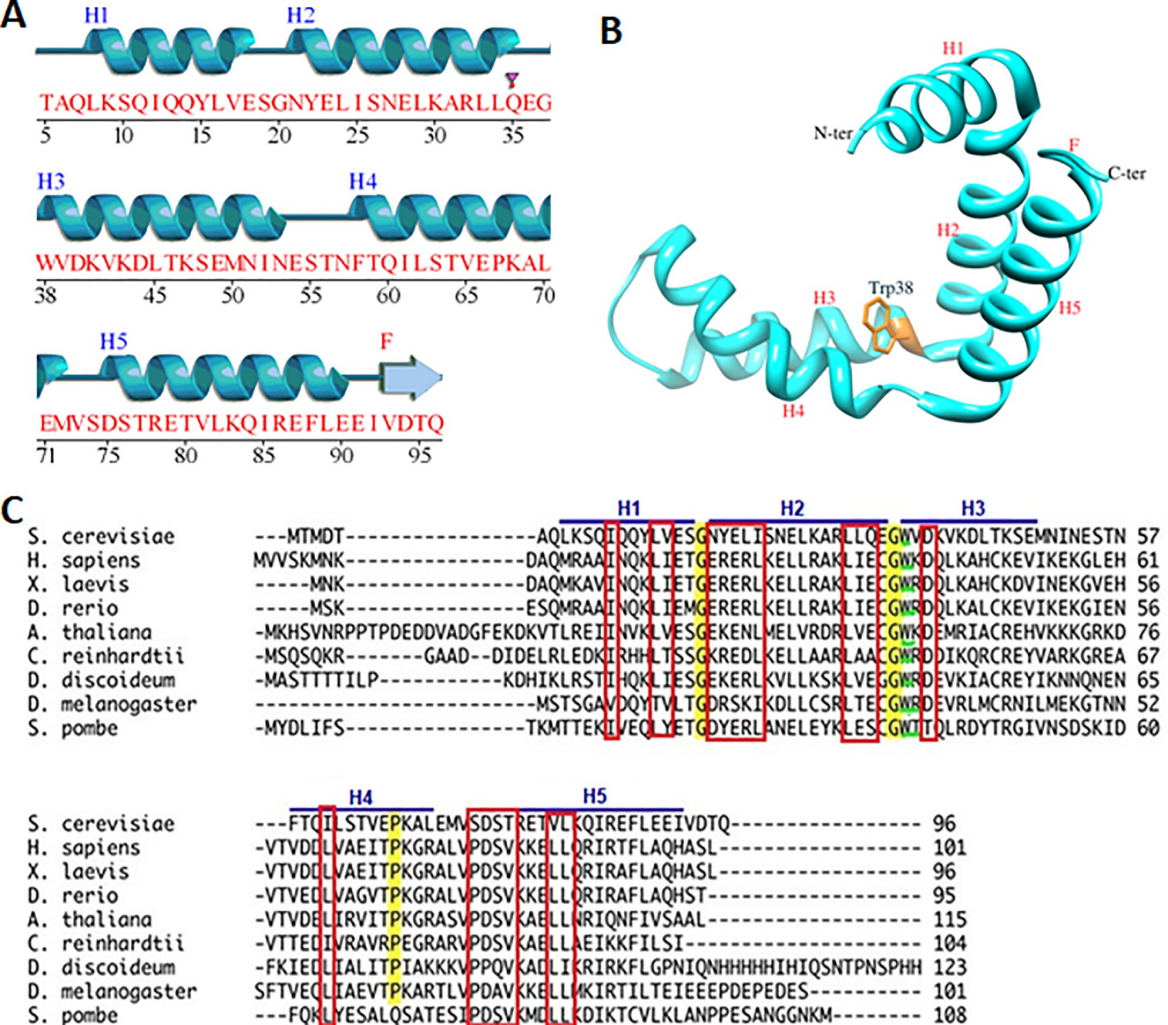
Sequence and structural analysis of Sus1. (A) Sequence along with the secondary structure was mapped using PDBsum server. H1- H5 represents the 5 helixes present in the Sus1. (B) The Sus1 structure was extracted from the PDB-3fwb using UCSF chimera. Helix and Trp38residue is labeled. (C) Multiple sequence alignment of the Sus1 from the different organism. The conserved residues are marked in the red rectangle. The conserved Gly residues between the H1-H2 and H2-H3 are highlighted in yellow color. The Pro67 residue is also highlighted in H4 helix. The conserved Trp residue is highlighted in green color.

**Fig 2:**
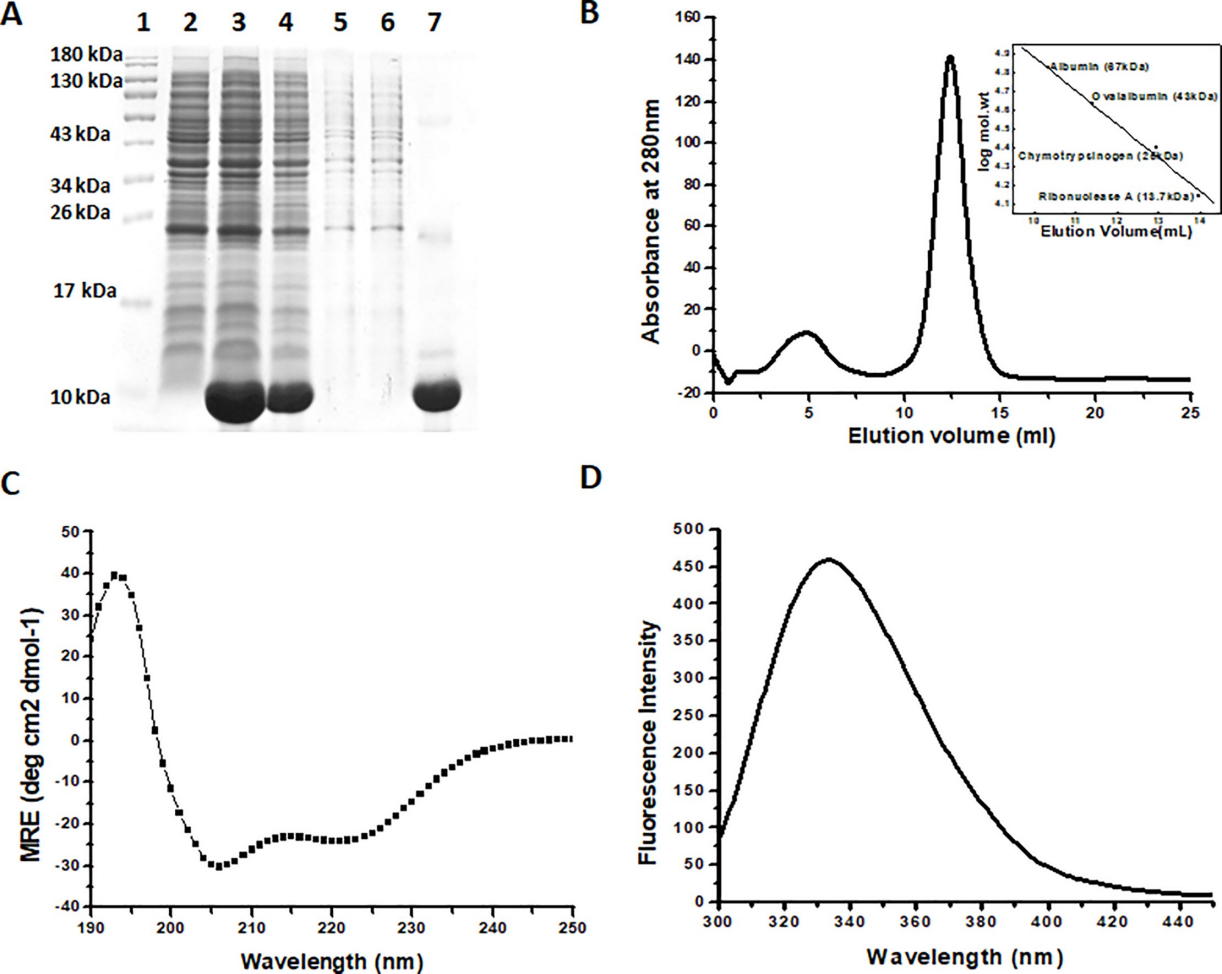
Purification and structural characterization of Sus1. (A) Denaturing gel representing the protein sample at different stages of the purification. Lane 1 represents the protein marker while 2,3,4,5,6,7 represent the uninduced cell lysate, induced cell lysate, soluble fraction of the cell lysate, washing fraction 1 (washing with equilibration buffer with 20 mM imidazole), washing fraction 2 (washing with equilibration buffer with 50 mM imidazole) and elution fraction (elution with equilibration buffer with 300 mM imidazole) respectively. (B) AKTA profile of purification of the Sus1 elution fraction 3 using SuperdexTM 75, 10/300GL column. Inset represents the standard plot between the log mol. weight and elution volume. The proteins taken to plot standard graph are 67KDa (Albumin), 43KDa (Ovaalbumin), 25KDa (Chymotrypsinogen) and 13.70KDa (Ribonuclease A). (C) CD spectra of the native protein represent two minima at 208 nm and 222 nm. (D) Fluorescence spectra of native protein.

Sus1 is a 96 amino acid long polypeptide with a molecular weight of 11.07 kDa. The structure analysis using PDBsum web server [25] depicts the presence of 5 α-helices (Fig 1A). The analysis of PDB:3FWB using UCSF chimera [26] shows the presence of one Trp residue in the vicinity which makes it perfect for the biophysical studies (Fig 1B). The multiple alignment result shows the presence of two conserved Gly residue at 20^th^ and 37^th^ position, have a role in helix turn. We also observed the presence of one conserved Pro residue at 67^th^ position (Fig 1C). The purification of the Sus1 has already been described in the method section. The Fig 2A represented the analysis of the protein sample at different purification steps where the protein was only present in the induced fraction (Fig 2A, lane 3) and a considerable amount of protein was there in the soluble fraction of the protein (Fig 2A, lane 4). The protein was further purified from the soluble fraction using the nickel NTA column. The purified protein was loaded on the denaturing acrylamide gel (SDS-PAGE) to check the homogeneity which is represented by a single protein band (Fig 2A, lane 7). The molecular mass of Sus1 under non-dissociating conditions using SEC (Size exclusion chromatography) was approximately 35 kDa (elution volume 12 ml), suggesting a trimeric state of the protein (Fig 2B) corresponding to standard plot (Fig 2B inset). The protein native state was checked using CD and fluorescence spectroscopy (Fig 2C and 2D). The CD analysis states two clear minima at 208 and 222 nm which shows a characteristic feature of α helix [22,27]. Furthermore, the native state of Sus1 exhibited emission maxima at 334 nm in fluorescence spectroscopy with excitation wavelength of 290 nm.

### pH dependent structural transition of Sus1

**Fig 3:**
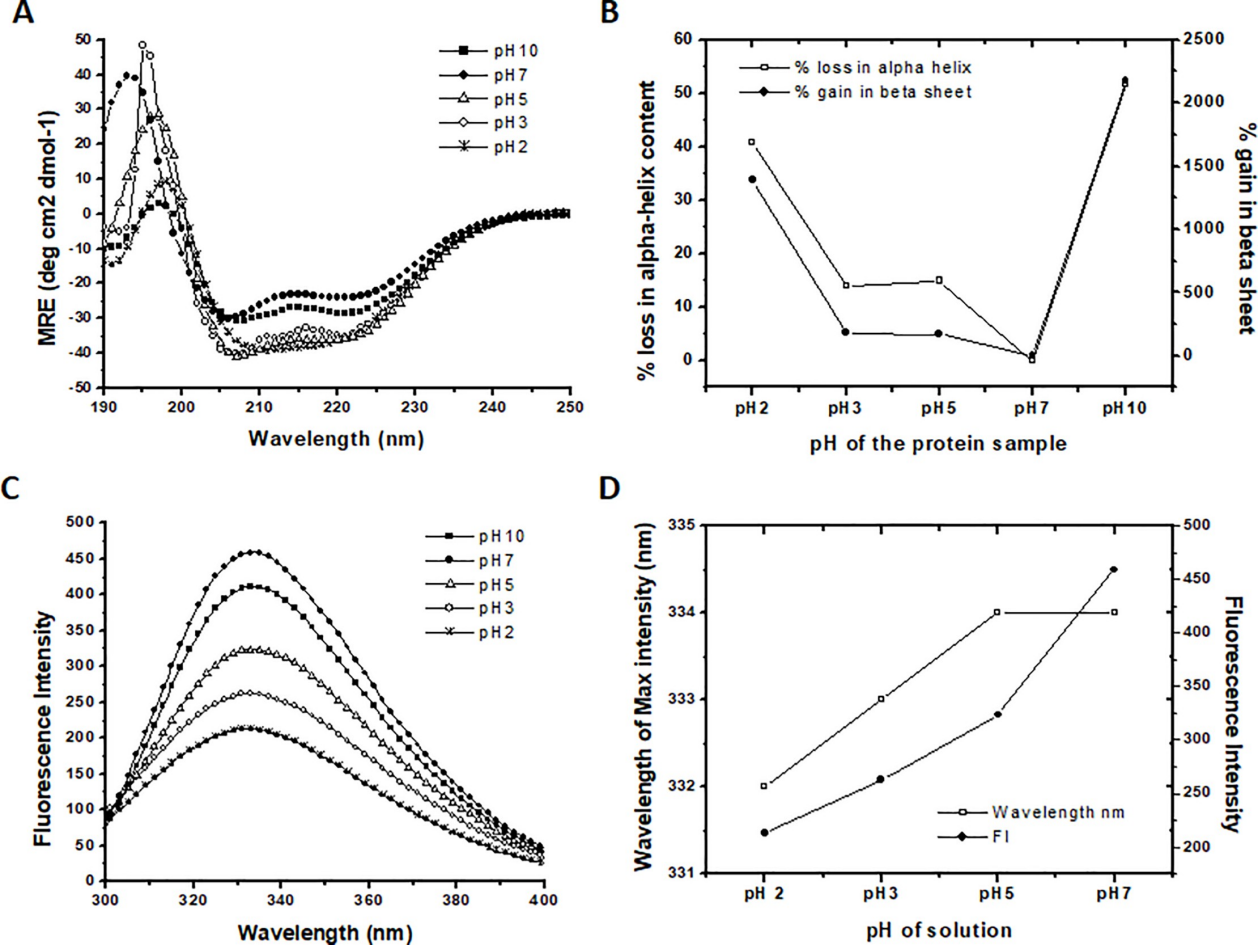
pH-dependent structural transition of Sus1. (A) Represent far-UV CD spectra, (B) represent the % loss of α-helix and % gain in β-sheets (C) represents the fluorescence emission spectrum and (D) represent the plot of fluorescence intensity and wavelength of maximum intensity at different pH. Native protein (pH-7) represented by closed circle (●) followed by protein samples at pH-2, 3, 5 and 10 represented by star (⁎), open circle (○), open triangle (Δ) and filled square (■) respectively. % loss of α-helix is represented by open square (□) and % gain in β-sheets are represented by closed circle (●). Similarly, the wavelength of maximum intensity and fluorescence intensity is represented by open square (□) and closed circle (●) respectively. The % α-helix and β-sheets were calculated using the K2D3 web server.

The secondary structure of the Sus1 was studied using the UV-CD spectrum, which showed Sus1 exist as a α-helical protein. Interestingly, from pH-7 to pH-3 and even at pH-10 the protein showed stability in the secondary structure (Fig 3A). Further decrease in pH (pH-2) resulted with single negative minima near 215 nm (Fig 3A). We investigated the decrease in the MRE values of222 nm and 208 nm from pH-10 to pH-5 as compared to pH-7, after that the MRE value of222 nm remained almost stable but slight increase in the value of 208 nm (S1 Fig). We compared the MRE value of pH-7 (native state) to that of various pH which showed decrease in the MRE value. The decrease in the MRE value as compared to native protein at pH-7 represented the stabilization of the secondary structure at low pH as well as at pH-10. Using the MRE value α-helix and β-sheet content was calculated using online server K2D3 (Table 1) [21]. Despite the stabilization of the secondary structure we observed the loss in the α-helix and gain in the β-sheet content at low as well as at high pH as compared to pH-7.At neutral pH (pH-7) Sus1 contained68.34% α-helix and 0.48% β-sheet, but as we drop down the pH till 3, loss in the α-helical content up to 58.84% and little increase in β-sheet content up to 1.35%were reported. At pH-2 α-helical content was further drop down to 40.41%, but increase in β-sheet content up to 7.15% (Table 1). We plotted the percentage decrease in α-helix and % increase in β-sheet content at different pH (Fig 3B). The results indicated ~40% loss in the α-helical content but there was more than ~1400% gain in the β-sheet content at pH-2 as compared to the native protein (Table 1, Fig 3B)[28]. The pH above 7 (pH-10) also showed the loss in the α-helical content more than ~50% and gain in the β-sheet content upto ~2200%.

**Table 1:** Showing α helix and β-sheet content in recombinant Sus1 at different pH. Percentage of α-helix and β-sheet content was calculated using the MRE value at (190nm-240nm) using online software K2D3.

pH of buffer	α-helix content	β-sheets content
2	40.41	7.15
3	58.84	1.35
5	58.12	1.29
7	68.34	0.48
10	32.97	10.93

After the secondary structure we studied the change in the tertiary structure by fluorescence spectroscopy. Sus1 contains single Trp residue which make Sus1 an ideal protein for biophysical studies. Native state of Sus1 exhibited emission maxima at 334 nm in fluorescence spectroscopy. As we decrease the pH from 7 to 2 the fluorescence intensity (FI) was reduced to ~50% (Fig 3C, Fig 3D) indicating a decrease in the polarity of the tryptophan microenvironment and thus increase in solvent accessibility to the core of the protein. The increase of the solvent accessibility represent that the Trp microenvironment has become non-polar.

### ANS fluorescence and acrylamide quenching show the accessibility of hydrophobic patches

**Fig 4:**
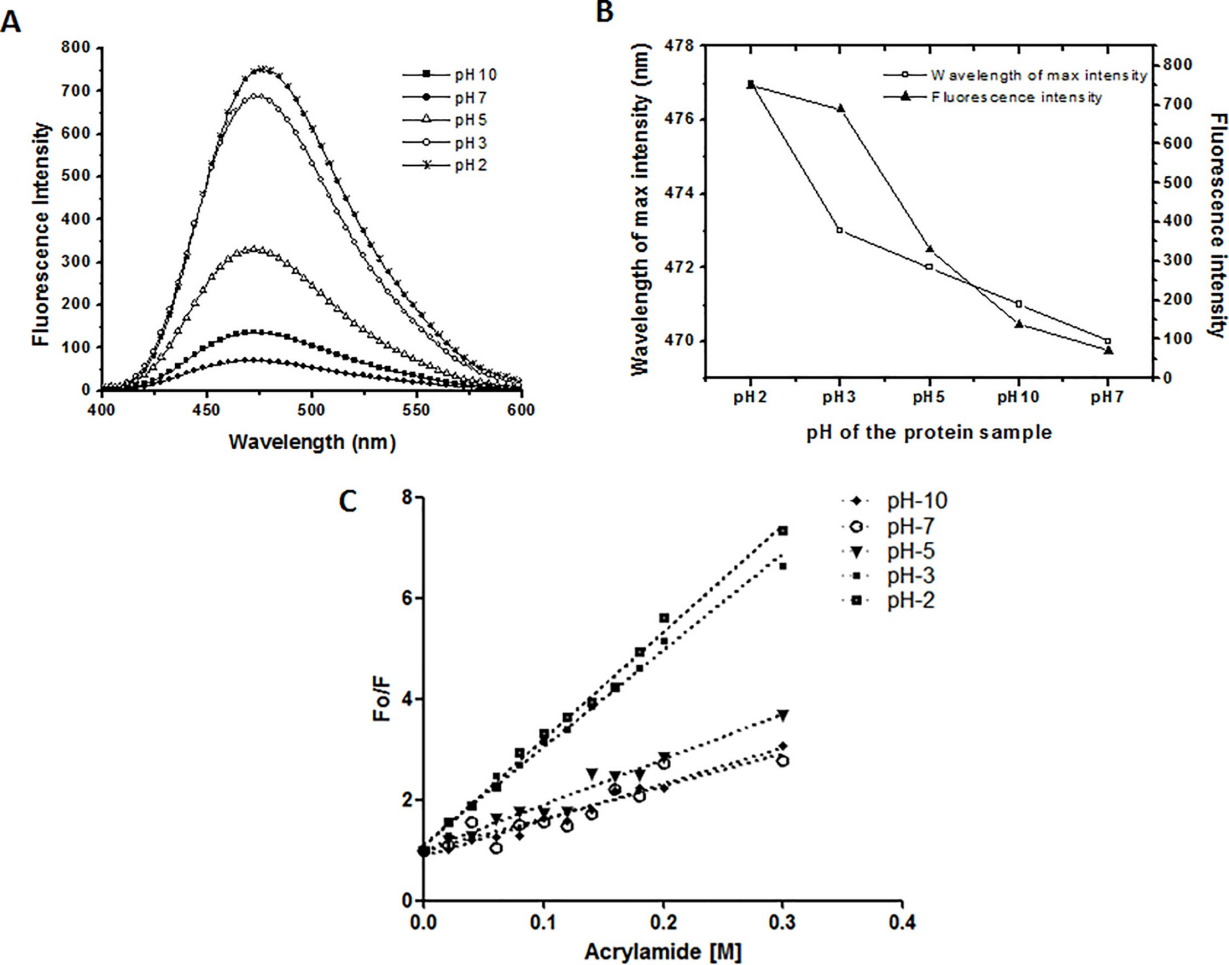
Effect of ANS and Acrylamide on the tertiary structure of Sus1. (A) ANS fluorescence spectra of Sus1 at different pH. Native protein (pH-7) represented by closed circle (●) followed by protein samples at pH-2, 3, 5 and 10 represented by star (⁎), open circle (○), open triangle (Δ) and filled square (■) respectively. (B) Graph represents the wavelength of maximum intensity and fluorescence intensity after the ANS treatment at different pH. The wavelength of maximum intensity and fluorescence intensity is represented by open square (□) and closed triangle (▲). (C) Represent the Stern-Volmer plot obtained from recombinant Sus1 fluorescence quenching at different pH using the different concentration of the acrylamide. The plot of native protein at pH-7, pH-10, pH-5, pH-3 and pH-2 are represented by open circle (○), closed diamond (♦), closed downward triangle (▼), small closed square (■) and bigger closed square (■). Protein concentration was 8 μM. The molar ratio of protein to ANS was 1:50.

A comparison of ANS fluorescence spectra of Sus1 at different pH was shown in Fig 4A. As expected, the spectra of native Sus1 showed negligible ANS fluorescence. ANS fluorescence was very much significant at pH 3.0 and pH 2.0 (Fig 4A). The fluorescence intensity and the wavelength of maximum intensity were plotted at different pH (Fig 4B). In the native protein the hydrophobic patches were buried because of that the ANS was unable to bind the protein, hence, observed low or negligible ANS fluorescence near neutral pH [27]. At the low pH the solution became acidic, which affected the tertiary structure of protein, which further, allowed the access of ANS molecules to the buried hydrophobic patches of protein [23,28]. The ANS can bind to the hydrophobic patches and positively charged residues in partially unfolded protein and ANS fluorescence get increased [23]. Similarly, at the higher pH-10 the protein again attended partially unfolded structure which also showed a little ANS fluorescence as compared to the native protein.

Acrylamide quenching is one of the popular methods to probe tryptophan environments in proteins [29]. Tryptophenyl fluorescence quenching study of the Sus1 in presence of different concentration of acrylamide was done at different pH and Stern-Volmer plot was made (Fig 4C). The slope of such plots is related to the degree of exposure (accessibility) of the tryptophan, which represent Stem-Volmer constant (Ksv). The Ksv and Kq value were calculated at different pH (Table 2). The Stern-Volmer constant (Ksv) of native Sus1 protein (pH-7) was found to be 6.47 M^−1^. However, the value of Ksv at pH-5 and pH-10 was 9.02 M^−1^ and 7.18 M^−1^ respectively almost comparable to the native protein. The Stern-Volmer plot indicated that the aromatic amino acids at pH-5 and pH-10 were only partially exposed as compared to the native folded conformation at pH-7. At pH-2 and pH-3 the Stern-Volmer constant (Ksv) was found to be 21.28 M^−1^ and 19.26 M^−1^ which represented that the structure was highly exposed at the low pH as compared to native protein. The results indicated at the lower pH the hydrophobic patches were partially exposed to bind with the ANS and acrylamide.

**Table 2:** Fluorescence quenching of Sus1 in presence of acrylamide at different pH states. The ratio of FI in the absence and presence of acrylamide at different concentration was plotted with the acrylamide concentration. Using this plot the Ksv and Kq were calculated.

pH of the protein sample	Ksv [M^-1^]	Kq [M^-1^s^-1^]
10	7.18	1.67 x 10^−9^
7	6.47	1.50 x 10^−9^
5	9.02	2.09 x 10^−9^
3	19.26	4.47 x 10^−9^
2	21.28	4.94 x 10^−9^

Furthermore, we asked the question whether the pH changes only affected the secondary and tertiary structure of the protein or it also affected the oligomerization status of the protein. We checked the oligomerization of protein by SEC using Superdex^TM^ 75 10/300 GL column at different pH. The results indicated the pH variation didn’t affect the oligomerization status of the protein (S2 Fig).

### Ethanol and ammonium sulfate stabilizes the secondary structure of Sus1

**Fig 5:**
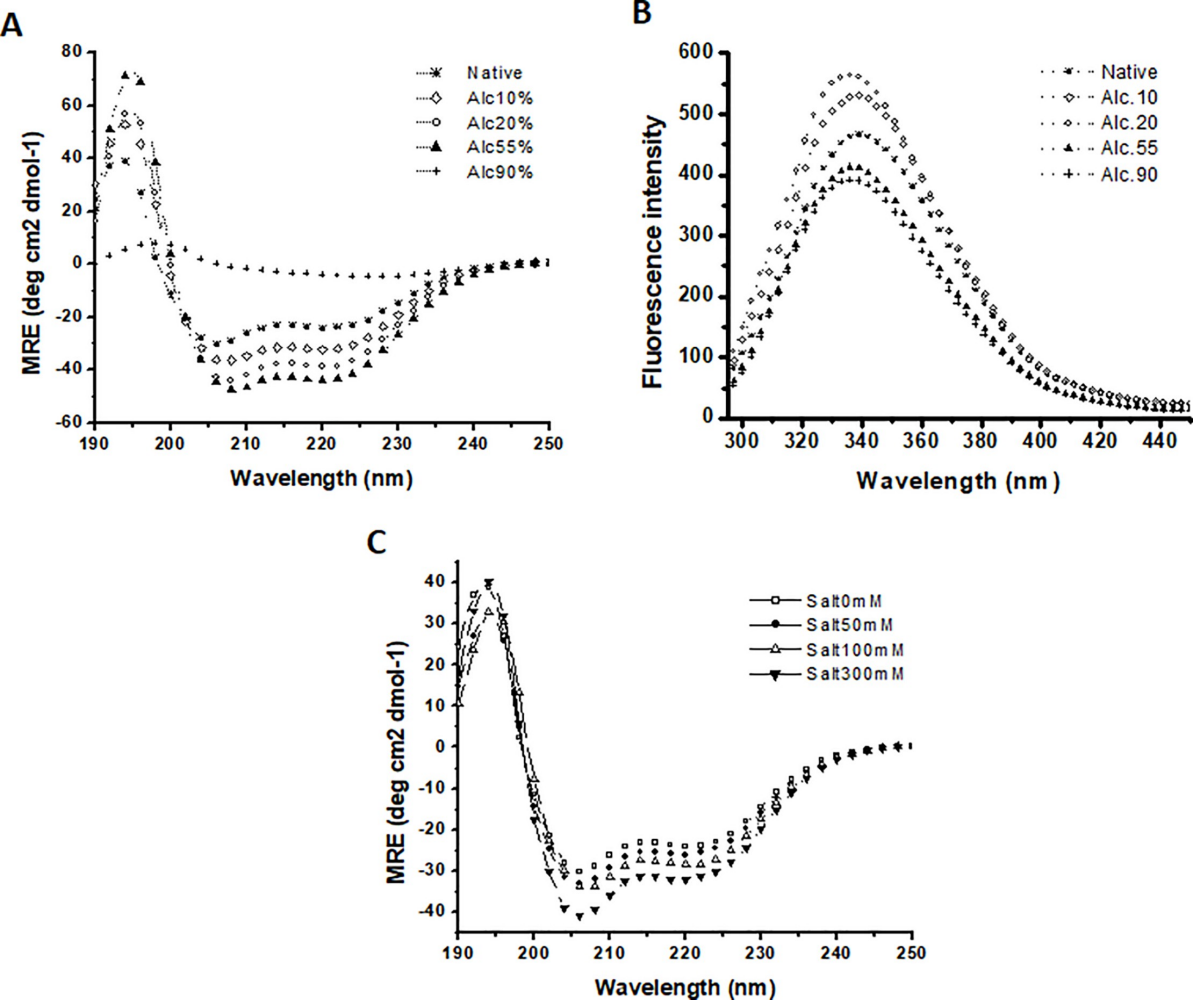
Ethanol and ammonium sulfate stabilizes the secondary structure of Sus1. (A) Far UV CD spectra in the absence and presence of different ethanol concentrations. (B) Fluorescence intensities of Sus1 native in absence and in presence of 10, 20, 50 and 90% ethanol concentrations. The plot of native protein at pH-7 in presence of 0, 10, 20, 50 and 90% ethanol is represented by star (⁎), open diamond (◊), open circle (○), close triangle (▲) and plus sign (+) respectively. (C) Far UV CD spectra in the absence (0mM) and in presence (50–300 mM) of ammonium sulphate at pH 7. The plot of native protein at pH-7 in absence and in presence of 0, 50, 100 and 300 mM ammonium sulfate is represented by open square (□), close circle (●), open tringle (Δ) and closed downward triangle (▼) respectively.

There are many studies which have revealed alcohols as a protein denaturants [30]. It can destruct the native structure of the protein. It has also ability as an inducer of α-helix/β-sheet as well as can dissolute the protein aggregates. Sus1 was found to get anchored with mega complexes like SAGA and TREX complexes, which depict Sus1 as quite flexible molecule. We checked the flexibility of Sus1 in the presence of ethanol by the far UV CD and tryptophenyl fluorescence spectroscopy to study the effect of the hydrophobic environment on the protein conformation. Fig 5A shows stabilization of the secondary structure in the presence of 0 to 55% alcohol. Further increment in the alcohol concentration led to the total disruption of secondary structure (Fig 5B). The fluorescence study also indicated the increase in the fluorescence intensity from 10–20% alcohol concentration. Further increase in the alcohol concentration (55–90%)leads to the decrease in the fluorescence intensity(Fig 5B).

Ammonium sulfate increases the protein solubility and stabilizes the protein till a particular salt concentration this is termed as the salting in. On the other hand, after a particular concentration it starts destabilizing the proteins and start aggregation and protein get precipitated termed as salting out. We checked the effect of the different concentration of ammonium sulphate on Sus1 by measuring the tryptophenyl fluorescence using the far UV-CD at pH-7 (Fig 5C). We observed the increase in the α-helical structure from 0 to 300 mM salt concentration (Fig 5C) which concluded that increase in salt concentration stabilized its secondary structure.

## Discussion

Sus1 switches between the complexes and the compartment as well during the mRNA transport through gene gating [3,5]. The rigidness in the α-helix and its flexibility for the association with the different proteins makes the Sus1 important for the structural characterization [17]. In this manuscript we have revealed the structural changes in the Sus1 at different pH conditions, which can help us to better understand the Sus1 mode of action in mRNA export, nuclear export and mRNA biogenesis. Like other export factor (Mex67 and Yra1), Sus1 also undergoes structural reorientation at low pH ranges to attain a number of conformations during mRNA export in association with other proteins [9,17]. The biochemical and biophysical characterization of Sus1 has led us to know the conformation stability of Sus1 and also describe the behavior of the protein in different environments. For the first time, we describe here the structural characteristic features of single tryptophan containing transcription coactivator Sus1.The Sus1 is the part of the DUB module which consists of the Ubp8/Sus1/Sgf11/Sgf73 proteins [31]. This complex is highly conserved in the composition as well as functionally from yeast to humans. This complex is functionally associated with several human diseases like neurodegenerative spino cerebellar ataxia type 7 disease which is associated with the polyglutamin (polyQ) expansion in human Sgf73 [32,33]. Several subunits of the SAGA and ATXN7 also involved in pathogenesis of neurodegenerative diseases and Gcn5 deletion accelerates its progression [34,35]. Polyglutamic expansion causes the conformational changes in the β-sheet structure [32]. The structural and conformational analysis of these proteins may lead to the identification of new targets for the development of the therapeutic strategies for such diseases [32]. The SAGA complex is also reported to regulate gene transcription via interacting with the transcription factor Myc to regulate cell growth and survival. This close interaction with the transcription factor link SAGA to the array of human cancer driven by oncogenes [32,36]. Exploring the structural changes in different stages and dynamics of intermediates of folding pathway of Sus1 is necessary to understand the basic role of Sus1 in various aspects of gene regulation like mRNA export, nuclear export and RNA biogenesis in association with other transcription regulators TREX2 and SAGA complex.

The Sus1 is a properly folded α helical protein with 5 α-helixes, named H1 to H5 in the Fig 1A and 1B (H1 from residue 6–19, H2 from residue 21–36, H3 from residue 38–52, H4 from residue 58–71 and H5 from residue 75–92). These rigid α-helixes were separated by the flexible regions which were located between the helixes. The hinges between the H1-H2 and H2-H3 helix was made up of single Gly residue which was found to be conserved across the species (Fig 1C, highlighted in yellow color). The loop between the H3-H4 and H4-H5 contained 8 and 6 residues, respectively (Fig 1C). These two loops were variable between the species and found to be highly flexible. The Pro67 residue in the H4 introduced a kink (Fig 1A, highlighted in yellow color) which is conserved across species, probably help Sus1 to wrap around the various proteins during the mRNA export and nuclear transport [16,17].

The structural data indicated that the Sus1 had more than ~68% of α-helix structure. The same result was found in our secondary structure prediction (Table 1). The protein showed stability in the secondary structure, however significant loss in the α-helical content was observed in acidic as well as in basic pH (Fig 3B, Table 1). We observed the ~40% loss in the α-helical content at pH-2 while more than 50% loss at pH-10 (Table 1, Fig 3B). The native protein was observed with the clear minima at 208 and 222 nm along with the maxima at 194 nm. The alteration in the pH of the protein samples were observed with the change in the statistics of the minima at 208 and 222 nm. Along with the change in the minima statistics the shift in the peak of maxima near 194 nm was also observed. At pH-2 Sus1 behaved as β-sheet like protein (Fig 3A, single minima at 215 nm at pH-2) as we observed single minima at 215 nm. Despite minima there was maxima at 198 nm at pH-2 as well as at pH-10 which is the characteristics of the β-sheet protein. We also observed more than 14times or ~1400% (from 0.48% to 7.15%) increase in the β-sheets as compared to native protein (Fig 3B). Despite the loss in the α-helical content and β-sheet gain the graph clearly showed the overall gain in the secondary structure in acidic as well as in basic pH as we observed decrease in the MRE value at 222 nm as well as 208 nm as compared to native protein which represented that the secondary structure was stabile. This type of structural transition has been already reported in many proteins. Some of the HIV’s proteins like gp120 show the same type of transition where, β-sheets get converted to the α-helix when virus bind to the host membrane protein CD4 [37,38].

Furthermore, using intrinsic fluorescence spectroscopy the loss in tertiary structure was monitored. The 290nm excitation wavelength was used for Sus1 and emission spectra were captured between 300–450 nm. The protein showed fluorescence emission maxima (λ_max_) at 334 nm in the native state (Fig 2D, Fig 3C). When we decrease the pH of the protein from 7 to 2 we noticed 50% reduction in the fluorescence intensity (Fig 3C, Fig 3D). pH-7 concomitant maximum fluorescence intensity (FI) indicating that Trp residue was maximally exposed to the polar environment (Fig 3C). These results indicated that initially the Trp residue was buried in the polar environment of the protein at the neutral pH, as we decrease the pH from 7 to 2 the Trp microenvironment became non-polar which leads to unfolding of the recombinant Sus1. Probably, because of the change in the ionic concentration in the solution perturbed the structure of the protein, which had fetched the hydrophobic residues in the non polar environment which further led to the unfolding of the protein at low pH [39]. This also represented the increase of the solvent accessibility to the core of the protein.

The ANS binding studies support the intrinsic fluorescence studies. The spectra of native Sus1 showed a very negligible ANS fluorescence (Fig 4A). This represented that the tertiary structure was properly folded which didn’t allow ANS molecule to access and bound to properly buried hydrophobic patches. At pH-10 there was little increase in the FIand ~50% increase at pH-2 (Fig 4B). We also observed in the red shift in the wavelength (7 nm) with decrease in the pH (Fig 4B). This red shift and the increase in the fluorescence intensity represent the unfolding of the protein [22,23]. To further validate our result, we did the acrylamide quenching experiment (Fig 4C). The ratio of the FI without acrylamide and with acrylamide was plotted against acrylamide concentration which is also known as Stem-Volmer plot. The Stern–Volmer plot indicates that the aromatic amino acids at pH-2 and pH-3 were more exposed as compared to the native folded tertiary conformation at pH-7. Combining intrinsic and ANS fluorescence in addition to acrylamide quenching studies we can say that the decrease in the pH forces the Trp residue along with other hydrophobic and positively charged residues accessible to the solvent which become available to ANS and acrylamide binding [24,28].

Alcohols are known to have very enormous effects upon proteins structure either by destruction of the rigid native structure, induction of alpha and beta-helices or by dissolution of peptide aggregates [30,38,40]. Sus1 showed stabilization of the secondary structure in the presence of 10 to 55% alcohol while tertiary structure was stable up to 20% alcohol concentration. We also observed the increase in the α-helical content up to 55% alcohol concentration. The result indicate the stabilization of the secondary structure till 55% alcohol concentration while tertiary structure was stable up to 20% alcohol concentration. Further increase at 50 and 90% alcohol concentration led to the destabilization of the tertiary structure. Proteins integrity was also affected by the salt concentration in milieu. Some of the salt at high concentrations around the protein environment, for example e.g. (NH_4_)_2_SO_4_, NaCl and NaSCN, etc, have a very deep impact on the conformational integrity of protein [30]. Sus1 secondary structure stabilized (gain of α helical structure) as salt concentration increased from 0 to 300 mM (Fig 5C). These results provided significant insights about the biophysical and structural plasticity of Sus1 protein. Since Sus1 is conserved among eukaryotes, similar regulatory structural plasticity, mechanisms, functions are likely to exist in humans.

## Supporting information

S1 File (DOCX)

S1 Fig pH-dependent alteration in MRE. Mean residue ellipticity value (MRE) of the far UV-CD of Sus1 at 222 nm and 208 nm was plotted at different pH. MRE value at 222 nm and 208 nm is represented by filled square (■) and open circle (○) respectively.
(DOCX)

S2 Fig pH changes don’t affect the oligomerization of Sus1. AKTA profile of Sus1 at different pH. AKTA profile at pH7, pH5 and pH2 is represented by the closed circle (●), closed tringle (▲) and open circle (○) respectively.
(DOCX)
