# Supplementary material for: An insight into structural plasticity and conformational transitions of transcriptional co-activator Sus1
Source: PLoS One. 2020 Mar 5;15(3):e0229216. doi: 10.1371/journal.pone.0229216 (PMC7058303; doi:10.1371/journal.pone.0229216)
Supplement: S1 File — (DOCX) [file pone.0229216.s001.docx]

**S1_raw_image**


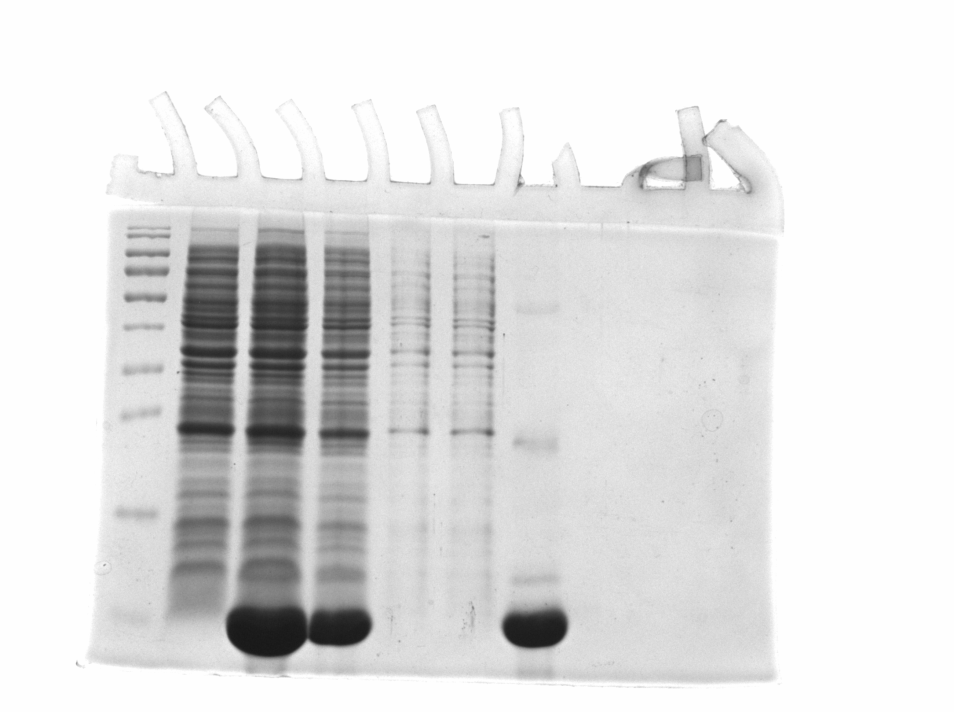


**S2_raw_table (CD at different pH)**

| Wavelength | pH2 | pH3 | pH5 | pH7 | pH10 |
| --- | --- | --- | --- | --- | --- |
| 190 | -13.5464 | -3.93822 | -8.65277 | 24.3131 | -10.073 |
| 191 | -14.6175 | -4.90847 | -4.26956 | 31.9759 | -9.54558 |
| 192 | -13.5136 | -5.13308 | 3.01889 | 36.9984 | -9.37281 |
| 193 | -9.67551 | -4.09918 | 10.6198 | 39.5116 | -6.8927 |
| 194 | -4.5328 | 12.6007 | 18.032 | 38.946 | -3.95974 |
| 195 | 0.63021 | 48.5493 | 24.1268 | 34.9234 | -0.64664 |
| 196 | 5.28164 | 45.2851 | 28.0614 | 26.9969 | 1.53401 |
| 197 | 8.438 | 27.5982 | 28.7771 | 14.9626 | 3.0455 |
| 198 | 9.21951 | 18.0913 | 24.551 | 2.36437 | 2.16377 |
| 199 | 7.07662 | 8.14061 | 16.7465 | -5.54758 | -0.19876 |
| 200 | 2.37878 | -4.56145 | 4.8433 | -11.5426 | -3.94067 |
| 201 | -4.09255 | -16.8634 | -7.6105 | -17.2903 | -8.86284 |
| 202 | -11.7366 | -25.651 | -18.6078 | -21.5064 | -14.5998 |
| 203 | -20.6422 | -31.0494 | -26.3175 | -24.9661 | -20.3131 |
| 204 | -26.7514 | -35.2046 | -32.2608 | -28.1721 | -25.7356 |
| 205 | -30.9525 | -38.9618 | -37.1847 | -29.8293 | -28.1804 |
| 206 | -34.2026 | -39.9135 | -39.9199 | -30.3064 | -30.0522 |
| 207 | -36.5607 | -40.0471 | -40.9578 | -29.8329 | -30.6294 |
| 208 | -37.8455 | -40.3302 | -40.7395 | -28.8995 | -30.8722 |
| 209 | -38.674 | -38.6941 | -39.9957 | -27.6264 | -30.0141 |
| 210 | -39.1545 | -36.4121 | -39.1233 | -26.2589 | -29.5191 |
| 211 | -39.2596 | -35.1872 | -37.884 | -25.0641 | -29.171 |
| 212 | -39.1359 | -35.645 | -37.0661 | -24.1496 | -28.4822 |
| 213 | -38.9017 | -35.2055 | -36.5483 | -23.5559 | -27.6081 |
| 214 | -38.6718 | -34.7794 | -36.11 | -23.1567 | -27.0699 |
| 215 | -38.3743 | -33.5349 | -35.9918 | -23.0196 | -26.9754 |
| 216 | -38.2481 | -32.9218 | -36.1581 | -23.2458 | -27.1672 |
| 217 | -38.1084 | -33.4522 | -36.305 | -23.4918 | -27.4453 |
| 218 | -37.6699 | -33.5547 | -36.3363 | -23.7064 | -27.8472 |
| 219 | -37.1156 | -34.401 | -36.3315 | -23.9167 | -28.2196 |
| 220 | -36.4957 | -34.9328 | -36.2875 | -24.1369 | -28.4686 |
| 221 | -35.7346 | -35.3422 | -35.9795 | -24.0913 | -28.5275 |
| 222 | -34.7853 | -34.6484 | -35.4026 | -23.9336 | -28.4155 |
| 223 | -33.382 | -32.6227 | -34.6357 | -23.6442 | -28.0449 |
| 224 | -31.7145 | -31.753 | -33.45 | -23.0651 | -27.3107 |
| 225 | -29.8544 | -31.2041 | -31.8007 | -22.2577 | -26.1503 |
| 226 | -27.8974 | -28.8319 | -29.9215 | -21.1643 | -24.7604 |
| 227 | -25.794 | -26.5083 | -27.877 | -19.6746 | -23.2293 |
| 228 | -23.5693 | -24.9049 | -25.5533 | -18.0587 | -21.5628 |
| 229 | -21.1707 | -22.7401 | -22.9914 | -16.3863 | -19.6839 |
| 230 | -18.6523 | -20.3245 | -20.3345 | -14.6411 | -17.6496 |
| 231 | -16.2338 | -18.2762 | -17.7917 | -12.8006 | -15.6159 |
| 232 | -13.9882 | -15.2377 | -15.3786 | -11.0246 | -13.6504 |
| 233 | -11.8369 | -12.1017 | -13.0723 | -9.32563 | -11.7809 |
| 234 | -9.88669 | -10.5583 | -11.0183 | -7.83906 | -10.0276 |
| 235 | -8.06098 | -8.65217 | -9.14289 | -6.50372 | -8.41061 |
| 236 | -6.46073 | -7.29479 | -7.40047 | -5.28497 | -6.94251 |
| 237 | -5.11624 | -5.9019 | -5.8445 | -4.24585 | -5.60755 |
| 238 | -4.03985 | -4.68072 | -4.5529 | -3.34135 | -4.53779 |
| 239 | -3.20956 | -4.09344 | -3.46569 | -2.59211 | -3.76466 |
| 240 | -2.54839 | -2.71372 | -2.5547 | -2.00481 | -3.0585 |
| 241 | -2.00753 | -2.00461 | -1.75341 | -1.50593 | -2.37109 |
| 242 | -1.47253 | -1.53009 | -1.0328 | -1.09628 | -1.83727 |
| 243 | -1.003 | -0.84814 | -0.51101 | -0.69667 | -1.46863 |
| 244 | -0.68037 | -0.96911 | -0.21792 | -0.40235 | -1.22264 |
| 245 | -0.44761 | -0.61633 | -0.05486 | -0.19364 | -1.02368 |
| 246 | -0.28485 | -0.21132 | 0.00102 | -0.02567 | -0.90853 |
| 247 | -0.12188 | -0.12147 | 0.04997 | 0.1079 | -0.79194 |
| 248 | -0.00832 | -0.20894 | 0.07381 | 0.23284 | -0.69172 |
| 249 | 0.05658 | -0.01001 | 0.06926 | 0.32116 | -0.63365 |
| 250 | 0.11827 | 0.18073 | 0.09093 | 0.34323 | -0.58564 |

**S3_raw_table (Intrinsic fluorescence at different pH)**

| Wavelength | pH2 | pH3 | pH5 | pH7 | pH10 |
| --- | --- | --- | --- | --- | --- |
| 300 | 82.824 | 95.082 | 87.446 | 83.595 | 77.098 |
| 301 | 88.252 | 102.252 | 95.332 | 94.592 | 86.978 |
| 302 | 93.929 | 109.337 | 103.527 | 106.435 | 97.821 |
| 303 | 99.36 | 116.23 | 111.808 | 118.953 | 109.233 |
| 304 | 104.939 | 123.274 | 120.554 | 132.343 | 121.463 |
| 305 | 110.505 | 130.128 | 129.526 | 146.607 | 133.893 |
| 306 | 116.082 | 137.115 | 138.843 | 161.159 | 147.244 |
| 307 | 121.93 | 144.017 | 148.565 | 176.763 | 160.628 |
| 308 | 127.783 | 150.688 | 158.002 | 192.462 | 174.29 |
| 309 | 133.432 | 157.898 | 167.782 | 207.798 | 188.641 |
| 310 | 138.959 | 165.085 | 177.714 | 222.981 | 202.967 |
| 311 | 144.632 | 171.889 | 187.29 | 238.425 | 217.329 |
| 312 | 149.924 | 178.489 | 196.878 | 254.21 | 231.329 |
| 313 | 155.158 | 185.182 | 206.525 | 269.889 | 245.645 |
| 314 | 160.426 | 191.584 | 215.91 | 285.674 | 259.172 |
| 315 | 165.066 | 198.218 | 224.934 | 301.231 | 272.881 |
| 316 | 169.747 | 204.266 | 233.738 | 316.224 | 286.106 |
| 317 | 174.31 | 210.668 | 242.384 | 330.808 | 298.759 |
| 318 | 178.639 | 216.418 | 251.291 | 345.419 | 311.518 |
| 319 | 182.477 | 221.775 | 259.586 | 359.076 | 323.441 |
| 320 | 186.286 | 226.986 | 267.725 | 372.179 | 334.775 |
| 321 | 189.805 | 231.694 | 275.627 | 384.991 | 345.482 |
| 322 | 193.27 | 236.576 | 282.872 | 396.616 | 355.86 |
| 323 | 196.535 | 240.562 | 289.715 | 407.788 | 364.847 |
| 324 | 200.002 | 244.35 | 295.621 | 417.554 | 373.539 |
| 325 | 202.766 | 248.044 | 301.315 | 425.765 | 381.82 |
| 326 | 205.287 | 251.105 | 306.367 | 432.871 | 388.422 |
| 327 | 207.416 | 253.894 | 310.803 | 439.304 | 394.184 |
| 328 | 209.214 | 256.492 | 314.918 | 445.122 | 399.317 |
| 329 | 211.059 | 258.502 | 318.087 | 449.599 | 403.56 |
| 330 | 212.194 | 260.306 | 320.348 | 453.372 | 406.754 |
| 331 | 212.751 | 261.442 | 322.049 | 455.975 | 409.178 |
| 332 | 213.342 | 262.16 | 322.996 | 457.918 | 410.517 |
| 333 | 213.155 | 262.57 | 323.342 | 459.098 | 411.316 |
| 334 | 212.445 | 262.112 | 323.416 | 459.399 | 411.466 |
| 335 | 211.541 | 261.101 | 322.757 | 458.455 | 410.424 |
| 336 | 210.696 | 260.127 | 321.671 | 456.247 | 408.674 |
| 337 | 209.162 | 258.387 | 320.45 | 453.565 | 406.583 |
| 338 | 207.477 | 256.49 | 318.483 | 449.933 | 404.194 |
| 339 | 205.514 | 254.106 | 316.389 | 445.545 | 400.487 |
| 340 | 203.539 | 251.876 | 313.925 | 440.348 | 396.318 |
| 341 | 200.853 | 249.059 | 311.223 | 434.854 | 391.842 |
| 342 | 198.085 | 246.588 | 307.996 | 428.58 | 386.583 |
| 343 | 195.112 | 244.231 | 304.627 | 422.276 | 380.93 |
| 344 | 192.286 | 241.31 | 300.759 | 415.549 | 375.191 |
| 345 | 189.556 | 238.269 | 296.1 | 408.086 | 368.568 |
| 346 | 186.203 | 234.973 | 291.489 | 400.824 | 361.796 |
| 347 | 182.739 | 231.229 | 286.364 | 393.209 | 354.507 |
| 348 | 179.409 | 227.318 | 280.799 | 385.516 | 347.149 |
| 349 | 175.67 | 223.276 | 275.791 | 377.479 | 340.401 |
| 350 | 172.085 | 218.898 | 270.369 | 369.917 | 332.614 |
| 351 | 168.363 | 214.644 | 264.815 | 361.824 | 324.554 |
| 352 | 164.882 | 210.353 | 258.579 | 353.302 | 316.779 |
| 353 | 160.912 | 205.36 | 252.987 | 344.651 | 309.1 |
| 354 | 157.114 | 200.873 | 247.206 | 336.114 | 301.397 |
| 355 | 152.978 | 196.245 | 241.604 | 327.169 | 293.229 |
| 356 | 149.006 | 191.434 | 236.031 | 318.039 | 285.051 |
| 357 | 145.106 | 186.545 | 229.985 | 309.051 | 277.115 |
| 358 | 141.033 | 182.274 | 223.628 | 299.942 | 269.179 |
| 359 | 137.118 | 177.697 | 217.27 | 291.028 | 260.997 |
| 360 | 133.306 | 172.78 | 211.147 | 281.675 | 253.187 |
| 361 | 129.685 | 168.191 | 204.817 | 272.161 | 245.011 |
| 362 | 125.776 | 163.22 | 198.719 | 262.808 | 236.922 |
| 363 | 121.801 | 158.387 | 192.637 | 253.679 | 228.722 |
| 364 | 118.077 | 153.638 | 186.775 | 244.925 | 220.88 |
| 365 | 114.306 | 148.877 | 181.18 | 236.567 | 213.85 |
| 366 | 110.577 | 144.03 | 175.085 | 228.587 | 205.872 |
| 367 | 106.644 | 139.358 | 169.231 | 220.517 | 198.64 |
| 368 | 103.119 | 134.988 | 163.299 | 212.91 | 191.861 |
| 369 | 99.64 | 130.638 | 158.014 | 205.242 | 185.178 |
| 370 | 96.262 | 126.835 | 152.457 | 197.979 | 178.614 |
| 371 | 92.849 | 122.813 | 147.428 | 190.65 | 172.257 |
| 372 | 89.741 | 118.716 | 142.529 | 183.183 | 165.95 |
| 373 | 86.646 | 114.869 | 137.319 | 176.454 | 159.821 |
| 374 | 83.325 | 111.191 | 132.353 | 170.185 | 154.04 |
| 375 | 80.383 | 107.163 | 127.678 | 163.905 | 148.401 |
| 376 | 77.418 | 103.448 | 123.292 | 157.627 | 142.867 |
| 377 | 74.61 | 99.63 | 118.744 | 151.317 | 137.129 |
| 378 | 71.731 | 95.915 | 114.466 | 145.313 | 131.483 |
| 379 | 68.8 | 92.295 | 110.201 | 139.298 | 125.846 |
| 380 | 66.094 | 88.581 | 105.909 | 133.622 | 120.341 |
| 381 | 63.307 | 84.712 | 101.753 | 127.752 | 115.371 |
| 382 | 60.557 | 81.158 | 97.479 | 122.113 | 110.063 |
| 383 | 57.762 | 77.633 | 93.622 | 116.544 | 105.294 |
| 384 | 55.289 | 74.478 | 89.35 | 111.067 | 100.372 |
| 385 | 52.924 | 71.182 | 85.024 | 105.722 | 95.805 |
| 386 | 50.406 | 67.964 | 80.868 | 100.425 | 91.198 |
| 387 | 48.078 | 64.769 | 77.1 | 95.164 | 86.787 |
| 388 | 45.847 | 61.754 | 73.425 | 90.193 | 82.489 |
| 389 | 43.607 | 58.801 | 69.573 | 85.686 | 78.337 |
| 390 | 41.478 | 55.928 | 66.149 | 81.295 | 74.376 |
| 391 | 39.665 | 53.257 | 62.912 | 76.941 | 70.535 |
| 392 | 37.721 | 50.81 | 59.769 | 73.026 | 66.866 |
| 393 | 35.916 | 48.232 | 56.98 | 68.959 | 63.171 |
| 394 | 34.159 | 46.022 | 54.103 | 65.469 | 59.674 |
| 395 | 32.376 | 43.836 | 51.542 | 61.784 | 56.371 |
| 396 | 30.873 | 41.876 | 49.154 | 58.467 | 53.219 |
| 397 | 29.27 | 39.973 | 46.944 | 55.411 | 50.556 |
| 398 | 27.951 | 38.144 | 44.695 | 52.622 | 47.833 |
| 399 | 26.665 | 36.369 | 42.869 | 49.957 | 45.346 |
| 400 | 25.481 | 34.935 | 40.874 | 47.645 | 43.03 |
| 401 | 24.318 | 33.33 | 39.032 | 45.336 | 41.04 |
| 402 | 23.156 | 31.87 | 37.517 | 43.122 | 39.126 |
| 403 | 22.301 | 30.635 | 36.118 | 41.13 | 37.409 |
| 404 | 21.359 | 29.484 | 34.763 | 39.377 | 35.841 |
| 405 | 20.541 | 28.341 | 33.435 | 37.82 | 34.513 |
| 406 | 19.895 | 27.317 | 32.277 | 36.355 | 33.239 |
| 407 | 19.276 | 26.395 | 31.094 | 34.912 | 32.013 |
| 408 | 18.51 | 25.424 | 29.984 | 33.685 | 30.847 |
| 409 | 17.91 | 24.578 | 28.968 | 32.404 | 29.633 |
| 410 | 17.274 | 23.768 | 27.865 | 31.398 | 28.555 |
| 411 | 16.61 | 23.049 | 27.026 | 30.175 | 27.566 |
| 412 | 16.163 | 22.412 | 26.158 | 29.117 | 26.662 |
| 413 | 15.658 | 21.638 | 25.34 | 28.327 | 25.798 |
| 414 | 15.254 | 20.982 | 24.448 | 27.487 | 24.832 |
| 415 | 14.837 | 20.393 | 23.744 | 26.569 | 23.963 |
| 416 | 14.347 | 19.817 | 22.99 | 25.605 | 23.097 |
| 417 | 13.981 | 19.128 | 22.23 | 24.638 | 22.268 |
| 418 | 13.622 | 18.66 | 21.554 | 23.744 | 21.508 |
| 419 | 13.264 | 18.121 | 20.935 | 22.961 | 20.824 |
| 420 | 12.896 | 17.641 | 20.358 | 22.123 | 20.257 |
| 421 | 12.613 | 17.152 | 19.799 | 21.289 | 19.703 |
| 422 | 12.371 | 16.656 | 19.282 | 20.454 | 19.096 |
| 423 | 12.016 | 16.205 | 18.703 | 19.661 | 18.435 |
| 424 | 11.66 | 15.718 | 18.137 | 18.916 | 17.808 |
| 425 | 11.436 | 15.331 | 17.501 | 18.327 | 17.161 |
| 426 | 11.218 | 14.86 | 16.974 | 17.69 | 16.525 |
| 427 | 11.008 | 14.526 | 16.524 | 17.104 | 16.006 |
| 428 | 10.817 | 14.201 | 16.081 | 16.539 | 15.507 |
| 429 | 10.512 | 13.846 | 15.565 | 16.054 | 14.936 |
| 430 | 10.357 | 13.516 | 15.226 | 15.603 | 14.434 |
| 431 | 10.105 | 13.156 | 14.927 | 15.08 | 13.97 |
| 432 | 9.904 | 12.864 | 14.564 | 14.651 | 13.448 |
| 433 | 9.796 | 12.688 | 14.23 | 14.229 | 12.958 |
| 434 | 9.684 | 12.395 | 13.965 | 13.854 | 12.603 |
| 435 | 9.499 | 12.091 | 13.79 | 13.447 | 12.263 |
| 436 | 9.4 | 11.893 | 13.451 | 13.079 | 11.985 |
| 437 | 9.247 | 11.649 | 13.16 | 12.643 | 11.61 |
| 438 | 9.116 | 11.424 | 12.865 | 12.314 | 11.243 |
| 439 | 8.982 | 11.254 | 12.45 | 11.938 | 10.935 |
| 440 | 8.848 | 10.982 | 12.095 | 11.65 | 10.676 |
| 441 | 8.761 | 10.788 | 11.768 | 11.321 | 10.376 |
| 442 | 8.674 | 10.621 | 11.45 | 10.889 | 10.08 |
| 443 | 8.674 | 10.621 | 11.45 | 10.889 | 10.08 |
| 444 | 8.674 | 10.621 | 11.45 | 10.889 | 10.08 |
| 445 | 8.674 | 10.621 | 11.45 | 10.889 | 10.08 |
| 446 | 8.674 | 10.621 | 11.45 | 10.889 | 10.08 |
| 447 | 8.674 | 10.621 | 11.45 | 10.889 | 10.08 |
| 448 | 8.674 | 10.621 | 11.45 | 10.889 | 10.08 |
| 449 | 8.674 | 10.621 | 11.45 | 10.889 | 10.08 |
| 450 | 8.674 | 10.621 | 11.45 | 10.889 | 10.08 |

**S4_raw_table (ANS Fluorescence)**

| Wavelength | pH2 | pH3 | pH5 | pH7 | pH10 |
| --- | --- | --- | --- | --- | --- |
| 400 | 8.902 | 10.897 | 9.592 | 3.343 | 4.098 |
| 401 | 8.902 | 10.897 | 9.592 | 3.343 | 4.098 |
| 402 | 8.902 | 10.897 | 9.592 | 3.343 | 4.098 |
| 403 | 8.902 | 10.897 | 9.592 | 3.343 | 4.098 |
| 404 | 8.902 | 10.897 | 9.592 | 3.343 | 4.098 |
| 405 | 8.902 | 10.897 | 9.592 | 3.343 | 4.098 |
| 406 | 8.902 | 10.897 | 9.592 | 3.343 | 4.098 |
| 407 | 8.902 | 10.897 | 9.592 | 3.343 | 4.098 |
| 408 | 8.902 | 10.897 | 9.592 | 3.343 | 4.098 |
| 409 | 10.419 | 12.604 | 10.585 | 3.678 | 4.562 |
| 410 | 12.184 | 14.564 | 11.751 | 4.082 | 5.07 |
| 411 | 14.215 | 16.896 | 13.071 | 4.495 | 5.629 |
| 412 | 16.566 | 19.577 | 14.698 | 4.953 | 6.322 |
| 413 | 19.191 | 22.757 | 16.519 | 5.463 | 7.056 |
| 414 | 22.332 | 26.328 | 18.531 | 6.017 | 7.953 |
| 415 | 25.949 | 30.232 | 20.87 | 6.624 | 8.941 |
| 416 | 29.939 | 34.559 | 23.431 | 7.295 | 10.022 |
| 417 | 34.428 | 39.445 | 26.184 | 8.054 | 11.181 |
| 418 | 39.315 | 44.976 | 29.171 | 8.863 | 12.477 |
| 419 | 44.848 | 51.108 | 32.45 | 9.699 | 13.912 |
| 420 | 50.899 | 57.76 | 36.133 | 10.608 | 15.488 |
| 421 | 57.656 | 64.998 | 40.07 | 11.575 | 17.114 |
| 422 | 64.971 | 72.907 | 44.343 | 12.66 | 18.92 |
| 423 | 72.986 | 81.457 | 49.049 | 13.847 | 20.869 |
| 424 | 81.708 | 90.681 | 54.098 | 15.057 | 22.952 |
| 425 | 91.1 | 100.417 | 59.557 | 16.35 | 25.207 |
| 426 | 101.424 | 111.162 | 65.273 | 17.744 | 27.56 |
| 427 | 112.346 | 122.804 | 71.448 | 19.163 | 30.065 |
| 428 | 124.059 | 134.972 | 77.921 | 20.703 | 32.782 |
| 429 | 136.463 | 147.88 | 84.675 | 22.345 | 35.585 |
| 430 | 149.706 | 161.427 | 91.643 | 23.951 | 38.448 |
| 431 | 163.538 | 175.325 | 99.034 | 25.637 | 41.566 |
| 432 | 177.873 | 189.702 | 106.563 | 27.355 | 44.649 |
| 433 | 192.735 | 204.824 | 114.178 | 29.103 | 47.817 |
| 434 | 208.199 | 220.657 | 121.98 | 30.825 | 51.115 |
| 435 | 224.547 | 236.81 | 130.117 | 32.556 | 54.344 |
| 436 | 241.428 | 253.283 | 138.301 | 34.319 | 57.7 |
| 437 | 258.597 | 270.111 | 146.569 | 36.09 | 61.082 |
| 438 | 275.931 | 287.377 | 154.914 | 37.913 | 64.433 |
| 439 | 293.691 | 304.52 | 163.246 | 39.656 | 67.795 |
| 440 | 312.026 | 321.417 | 171.593 | 41.283 | 71.196 |
| 441 | 330.426 | 338.732 | 179.934 | 42.876 | 74.706 |
| 442 | 349.177 | 356.14 | 188.068 | 44.456 | 78.035 |
| 443 | 367.907 | 373.417 | 196.377 | 46.016 | 81.364 |
| 444 | 386.699 | 390.709 | 204.548 | 47.585 | 84.739 |
| 445 | 405.312 | 407.915 | 212.736 | 49.101 | 88.007 |
| 446 | 423.679 | 424.764 | 220.671 | 50.577 | 91.08 |
| 447 | 441.851 | 441.332 | 228.412 | 52.041 | 94.286 |
| 448 | 460.027 | 457.795 | 235.925 | 53.335 | 97.239 |
| 449 | 477.824 | 474.209 | 243.237 | 54.76 | 100.222 |
| 450 | 495.701 | 490.266 | 250.492 | 56.09 | 103.034 |
| 451 | 513.398 | 506.22 | 257.661 | 57.409 | 105.779 |
| 452 | 530.709 | 521.872 | 264.448 | 58.725 | 108.56 |
| 453 | 547.582 | 536.725 | 270.833 | 59.97 | 111.18 |
| 454 | 564.042 | 551.243 | 276.869 | 61.192 | 113.636 |
| 455 | 580.053 | 564.517 | 282.661 | 62.205 | 116.155 |
| 456 | 595.634 | 577.264 | 288.267 | 63.149 | 118.548 |
| 457 | 610.208 | 589.611 | 293.468 | 64.075 | 120.789 |
| 458 | 624.555 | 600.873 | 298.266 | 64.969 | 122.885 |
| 459 | 638.031 | 611.668 | 302.966 | 65.75 | 124.817 |
| 460 | 650.671 | 621.7 | 307.33 | 66.521 | 126.547 |
| 461 | 662.48 | 630.738 | 311.135 | 67.245 | 128.074 |
| 462 | 673.616 | 639.14 | 314.657 | 67.956 | 129.359 |
| 463 | 684.383 | 647.34 | 317.719 | 68.402 | 130.715 |
| 464 | 694.753 | 654.842 | 320.391 | 68.858 | 131.944 |
| 465 | 703.89 | 661.368 | 322.746 | 69.395 | 133.087 |
| 466 | 712.656 | 667.353 | 324.806 | 69.72 | 134.135 |
| 467 | 720.568 | 673.002 | 326.516 | 70.136 | 135.032 |
| 468 | 727.376 | 677.537 | 327.913 | 70.474 | 135.693 |
| 469 | 732.995 | 681.115 | 328.709 | 70.643 | 136.193 |
| 470 | 737.578 | 684.289 | 329.471 | 70.723 | 136.604 |
| 471 | 741.205 | 686.299 | 329.692 | 70.711 | 136.866 |
| 472 | 744.053 | 688.05 | 329.701 | 70.624 | 136.797 |
| 473 | 746.299 | 688.861 | 329.469 | 70.517 | 136.728 |
| 474 | 748.441 | 688.823 | 328.64 | 70.275 | 136.542 |
| 475 | 749.909 | 688.141 | 327.781 | 69.935 | 136.2 |
| 476 | 750.522 | 686.635 | 326.656 | 69.65 | 135.753 |
| 477 | 750.777 | 684.598 | 325.201 | 69.328 | 135.211 |
| 478 | 750.113 | 682.709 | 323.683 | 68.866 | 134.572 |
| 479 | 748.935 | 680.374 | 321.48 | 68.349 | 133.926 |
| 480 | 747.362 | 677.179 | 319.111 | 67.888 | 133.346 |
| 481 | 745.112 | 673.111 | 316.82 | 67.322 | 132.552 |
| 482 | 742.345 | 668.653 | 314.302 | 66.82 | 131.781 |
| 483 | 738.761 | 663.674 | 311.412 | 66.403 | 130.84 |
| 484 | 734.324 | 657.997 | 308.604 | 65.927 | 129.857 |
| 485 | 728.828 | 652.131 | 305.458 | 65.376 | 128.667 |
| 486 | 723.134 | 645.694 | 302.334 | 64.866 | 127.422 |
| 487 | 716.835 | 638.987 | 298.756 | 64.342 | 126.024 |
| 488 | 710.578 | 632.509 | 295.392 | 63.636 | 124.574 |
| 489 | 703.851 | 625.624 | 291.769 | 63.016 | 123.217 |
| 490 | 697.392 | 618.327 | 287.921 | 62.32 | 121.758 |
| 491 | 690.184 | 610.568 | 284.166 | 61.549 | 120.297 |
| 492 | 682.298 | 602.644 | 280.224 | 60.783 | 118.792 |
| 493 | 674.252 | 594.473 | 276.055 | 60.039 | 117.229 |
| 494 | 665.638 | 586.575 | 271.738 | 59.214 | 115.573 |
| 495 | 656.814 | 578.175 | 267.259 | 58.458 | 113.905 |
| 496 | 648.096 | 569.486 | 262.823 | 57.618 | 112.379 |
| 497 | 639.155 | 560.288 | 258.526 | 56.741 | 110.675 |
| 498 | 629.693 | 550.946 | 254.032 | 55.991 | 109.039 |
| 499 | 620.439 | 541.416 | 249.395 | 55.233 | 107.382 |
| 500 | 610.922 | 531.926 | 244.989 | 54.496 | 105.677 |
| 501 | 600.672 | 522.366 | 240.572 | 53.693 | 103.807 |
| 502 | 590.823 | 512.714 | 235.967 | 52.826 | 102.108 |
| 503 | 580.951 | 503.131 | 231.524 | 51.895 | 100.354 |
| 504 | 570.836 | 493.808 | 226.994 | 50.9 | 98.652 |
| 505 | 560.625 | 484.338 | 222.565 | 49.99 | 96.89 |
| 506 | 550.608 | 474.807 | 218.075 | 49.023 | 95.043 |
| 507 | 539.94 | 465.169 | 213.596 | 48.091 | 93.277 |
| 508 | 529.644 | 455.561 | 208.97 | 47.174 | 91.654 |
| 509 | 519.32 | 445.974 | 204.395 | 46.408 | 89.948 |
| 510 | 509.319 | 436.854 | 200.034 | 45.569 | 88.223 |
| 511 | 499.125 | 427.703 | 195.724 | 44.789 | 86.538 |
| 512 | 489.134 | 418.59 | 191.345 | 43.988 | 84.914 |
| 513 | 479.279 | 409.338 | 187.164 | 43.178 | 83.169 |
| 514 | 469.347 | 400.281 | 182.951 | 42.44 | 81.501 |
| 515 | 459.786 | 391.094 | 178.681 | 41.721 | 79.763 |
| 516 | 450.007 | 382.197 | 174.607 | 40.994 | 78.043 |
| 517 | 440.124 | 373.382 | 170.603 | 40.272 | 76.326 |
| 518 | 430.511 | 364.586 | 166.44 | 39.482 | 74.746 |
| 519 | 421.176 | 355.75 | 162.643 | 38.749 | 73.102 |
| 520 | 411.421 | 347.368 | 158.498 | 38.037 | 71.489 |
| 521 | 402.197 | 339.089 | 154.559 | 37.317 | 69.914 |
| 522 | 392.775 | 330.989 | 150.583 | 36.621 | 68.311 |
| 523 | 383.325 | 322.856 | 146.773 | 35.893 | 66.864 |
| 524 | 374.136 | 314.987 | 142.99 | 35.16 | 65.373 |
| 525 | 365.345 | 307.422 | 139.341 | 34.487 | 63.961 |
| 526 | 356.764 | 300.082 | 136.022 | 33.731 | 62.541 |
| 527 | 348.063 | 292.596 | 132.67 | 33.026 | 61.175 |
| 528 | 339.601 | 285.309 | 129.374 | 32.323 | 59.902 |
| 529 | 331.434 | 278.1 | 126.004 | 31.579 | 58.595 |
| 530 | 323.321 | 270.975 | 122.759 | 30.892 | 57.318 |
| 531 | 315.488 | 263.964 | 119.593 | 30.245 | 55.915 |
| 532 | 307.622 | 257.175 | 116.657 | 29.615 | 54.631 |
| 533 | 299.962 | 250.756 | 113.713 | 28.969 | 53.375 |
| 534 | 292.438 | 244.314 | 110.769 | 28.357 | 52.101 |
| 535 | 285.242 | 238.094 | 107.918 | 27.824 | 50.869 |
| 536 | 278.052 | 231.942 | 105.008 | 27.304 | 49.66 |
| 537 | 270.984 | 225.727 | 102.133 | 26.738 | 48.489 |
| 538 | 264.099 | 219.669 | 99.372 | 26.196 | 47.284 |
| 539 | 257.177 | 213.686 | 96.669 | 25.597 | 46.106 |
| 540 | 250.505 | 207.879 | 94.047 | 25.058 | 44.929 |
| 541 | 244.001 | 201.979 | 91.403 | 24.491 | 43.81 |
| 542 | 237.21 | 196.024 | 88.966 | 23.948 | 42.691 |
| 543 | 230.617 | 190.375 | 86.36 | 23.431 | 41.643 |
| 544 | 224.207 | 184.771 | 83.833 | 22.898 | 40.625 |
| 545 | 217.898 | 179.262 | 81.229 | 22.304 | 39.549 |
| 546 | 211.721 | 173.817 | 78.778 | 21.825 | 38.445 |
| 547 | 205.883 | 168.531 | 76.416 | 21.237 | 37.369 |
| 548 | 199.918 | 163.237 | 74.151 | 20.698 | 36.33 |
| 549 | 194.045 | 158.432 | 71.779 | 20.171 | 35.304 |
| 550 | 187.854 | 153.376 | 69.51 | 19.708 | 34.285 |
| 551 | 181.744 | 148.519 | 67.286 | 19.201 | 33.318 |
| 552 | 175.732 | 143.553 | 65.118 | 18.688 | 32.316 |
| 553 | 169.688 | 138.663 | 62.941 | 18.155 | 31.321 |
| 554 | 163.955 | 133.905 | 60.905 | 17.621 | 30.333 |
| 555 | 158.097 | 129.207 | 58.866 | 17.081 | 29.36 |
| 556 | 152.426 | 124.476 | 56.895 | 16.579 | 28.4 |
| 557 | 146.825 | 119.932 | 54.893 | 16.077 | 27.514 |
| 558 | 141.428 | 115.331 | 52.893 | 15.6 | 26.645 |
| 559 | 136.226 | 111.142 | 50.927 | 15.106 | 25.759 |
| 560 | 130.985 | 106.794 | 49.005 | 14.579 | 24.883 |
| 561 | 125.967 | 102.551 | 47.098 | 14.093 | 23.988 |
| 562 | 121.064 | 98.151 | 45.137 | 13.659 | 23.091 |
| 563 | 116.464 | 93.974 | 43.27 | 13.173 | 22.214 |
| 564 | 111.729 | 89.959 | 41.391 | 12.744 | 21.339 |
| 565 | 106.968 | 86.205 | 39.49 | 12.323 | 20.514 |
| 566 | 102.178 | 82.326 | 37.794 | 11.826 | 19.645 |
| 567 | 97.671 | 78.707 | 36.09 | 11.381 | 18.756 |
| 568 | 93.298 | 75.149 | 34.458 | 10.938 | 17.921 |
| 569 | 89.002 | 71.71 | 32.877 | 10.526 | 17.142 |
| 570 | 84.802 | 68.425 | 31.375 | 10.151 | 16.402 |
| 571 | 80.792 | 65.314 | 29.963 | 9.782 | 15.709 |
| 572 | 77.155 | 62.284 | 28.662 | 9.431 | 15.047 |
| 573 | 73.675 | 59.617 | 27.409 | 9.056 | 14.467 |
| 574 | 70.389 | 56.851 | 26.201 | 8.693 | 13.849 |
| 575 | 67.199 | 54.411 | 25.077 | 8.316 | 13.297 |
| 576 | 64.171 | 51.886 | 23.979 | 7.965 | 12.746 |
| 577 | 61.361 | 49.491 | 22.959 | 7.673 | 12.271 |
| 578 | 58.592 | 47.1 | 21.95 | 7.352 | 11.802 |
| 579 | 56.029 | 45.057 | 21.01 | 7.07 | 11.316 |
| 580 | 53.566 | 43.158 | 20.138 | 6.76 | 10.806 |
| 581 | 51.099 | 41.344 | 19.271 | 6.493 | 10.358 |
| 582 | 48.847 | 39.515 | 18.454 | 6.237 | 9.894 |
| 583 | 46.631 | 37.731 | 17.618 | 6.02 | 9.488 |
| 584 | 44.594 | 36.097 | 16.799 | 5.793 | 9.125 |
| 585 | 42.569 | 34.394 | 16.028 | 5.59 | 8.777 |
| 586 | 40.689 | 32.887 | 15.314 | 5.402 | 8.442 |
| 587 | 38.976 | 31.473 | 14.657 | 5.224 | 8.134 |
| 588 | 37.357 | 30.115 | 13.993 | 5.015 | 7.841 |
| 589 | 35.751 | 28.889 | 13.405 | 4.828 | 7.551 |
| 590 | 34.318 | 27.592 | 12.834 | 4.662 | 7.276 |
| 591 | 32.893 | 26.389 | 12.327 | 4.49 | 7.013 |
| 592 | 31.599 | 25.148 | 11.846 | 4.324 | 6.747 |
| 593 | 30.285 | 24.081 | 11.362 | 4.177 | 6.505 |
| 594 | 28.967 | 23.047 | 10.932 | 4.017 | 6.244 |
| 595 | 27.768 | 22.147 | 10.496 | 3.863 | 5.968 |
| 596 | 26.581 | 21.292 | 10.097 | 3.73 | 5.708 |
| 597 | 25.44 | 20.459 | 9.702 | 3.606 | 5.49 |
| 598 | 24.402 | 19.588 | 9.315 | 3.479 | 5.276 |
| 599 | 23.374 | 18.722 | 8.957 | 3.337 | 5.089 |
| 600 | 22.428 | 17.976 | 8.585 | 3.217 | 4.88 |

**S5_raw_table (CD in presence of alcohol)**

| Wavelength | native | a10 | a20 | a55 | a90 |
| --- | --- | --- | --- | --- | --- |
| 250 | 0.34323 | 0.74399 | 0.20838 | 0.24531 | -0.5024 |
| 249 | 0.32116 | 0.72622 | 0.12815 | 0.1949 | -0.56031 |
| 248 | 0.23284 | 0.64544 | 0.02536 | 0.11736 | -0.60428 |
| 247 | 0.1079 | 0.50223 | -0.13238 | -0.00989 | -0.66046 |
| 246 | -0.02567 | 0.28141 | -0.30628 | -0.19648 | -0.78001 |
| 245 | -0.19364 | -0.00572 | -0.47838 | -0.44691 | -0.93018 |
| 244 | -0.40235 | -0.36265 | -0.75075 | -0.84244 | -1.089 |
| 243 | -0.69667 | -0.79576 | -1.16007 | -1.33812 | -1.26616 |
| 242 | -1.09628 | -1.2523 | -1.66536 | -1.99025 | -1.46264 |
| 241 | -1.50593 | -1.71633 | -2.27436 | -2.83413 | -1.66872 |
| 240 | -2.00481 | -2.27504 | -3.01092 | -3.8805 | -1.90075 |
| 239 | -2.59211 | -3.05491 | -3.98057 | -5.15802 | -2.14264 |
| 238 | -3.34135 | -4.02343 | -5.20244 | -6.68545 | -2.3845 |
| 237 | -4.24585 | -5.16328 | -6.67154 | -8.42027 | -2.63751 |
| 236 | -5.28497 | -6.57141 | -8.37143 | -10.3843 | -2.91654 |
| 235 | -6.50372 | -8.24726 | -10.3604 | -12.6408 | -3.21572 |
| 234 | -7.83906 | -10.1626 | -12.6379 | -15.1107 | -3.52381 |
| 233 | -9.32563 | -12.2602 | -15.047 | -17.7506 | -3.79021 |
| 232 | -11.0246 | -14.4818 | -17.6265 | -20.6456 | -4.04799 |
| 231 | -12.8006 | -16.7791 | -20.3232 | -23.6508 | -4.30595 |
| 230 | -14.6411 | -19.1637 | -23.0466 | -26.6837 | -4.48971 |
| 229 | -16.3863 | -21.4747 | -25.7605 | -29.7286 | -4.63711 |
| 228 | -18.0587 | -23.7008 | -28.3434 | -32.6682 | -4.73845 |
| 227 | -19.6746 | -25.8608 | -30.8216 | -35.3725 | -4.7726 |
| 226 | -21.1643 | -27.7643 | -33.0989 | -37.7319 | -4.76659 |
| 225 | -22.2577 | -29.2798 | -35.0438 | -39.6852 | -4.71785 |
| 224 | -23.0651 | -30.5173 | -36.5866 | -41.2763 | -4.65182 |
| 223 | -23.6442 | -31.4412 | -37.7647 | -42.5711 | -4.53901 |
| 222 | -23.9336 | -32.0106 | -38.5699 | -43.4692 | -4.36771 |
| 221 | -24.0913 | -32.1996 | -38.9331 | -43.9367 | -4.1968 |
| 220 | -24.1369 | -32.192 | -38.9309 | -44.0087 | -4.0274 |
| 219 | -23.9167 | -32.1339 | -38.7514 | -43.8605 | -3.86091 |
| 218 | -23.7064 | -31.9419 | -38.3577 | -43.5468 | -3.66546 |
| 217 | -23.4918 | -31.6303 | -37.951 | -43.1345 | -3.54507 |
| 216 | -23.2458 | -31.3673 | -37.5054 | -42.6981 | -3.39512 |
| 215 | -23.0196 | -31.3131 | -37.42 | -42.4246 | -3.20973 |
| 214 | -23.1567 | -31.4809 | -37.7373 | -42.3987 | -3.04997 |
| 213 | -23.5559 | -31.808 | -38.3156 | -42.8043 | -2.81913 |
| 212 | -24.1496 | -32.5724 | -39.2755 | -43.7086 | -2.55197 |
| 211 | -25.0641 | -33.5538 | -40.5472 | -45.0412 | -2.24119 |
| 210 | -26.2589 | -34.5546 | -41.9995 | -46.5225 | -1.8104 |
| 209 | -27.6264 | -35.522 | -43.3136 | -47.6512 | -1.40767 |
| 208 | -28.8995 | -36.3335 | -43.9962 | -47.7004 | -1.13125 |
| 207 | -29.8329 | -36.6203 | -43.9076 | -46.5993 | -0.73658 |
| 206 | -30.3064 | -36.0339 | -42.6966 | -44.4093 | 0.09417 |
| 205 | -29.8293 | -34.4694 | -39.9357 | -41.1948 | 0.81929 |
| 204 | -28.1721 | -31.5701 | -35.7107 | -36.2517 | 1.79388 |
| 203 | -24.9661 | -27.1334 | -29.6628 | -29.0678 | 3.09093 |
| 202 | -21.5064 | -21.7544 | -21.088 | -20.0265 | 5.20936 |
| 201 | -17.2903 | -14.0477 | -11.1033 | -9.16509 | 6.45358 |
| 200 | -11.5426 | -4.27435 | -0.318 | 3.82141 | 7.31482 |
| 199 | -5.54758 | 7.96146 | 12.0135 | 19.6053 | 7.77417 |
| 198 | 2.36437 | 22.6982 | 27.0927 | 38.1813 | 7.8764 |
| 197 | 14.9626 | 35.1923 | 41.8342 | 56.3245 | 7.65285 |
| 196 | 26.9969 | 45.4014 | 53.4124 | 68.8223 | 7.13873 |
| 195 | 34.9234 | 51.2428 | 58.093 | 73.6943 | 6.30485 |
| 194 | 38.946 | 52.8232 | 56.7008 | 71.3316 | 5.32628 |
| 193 | 39.5116 | 50.6476 | 50.4635 | 62.9575 | 4.15891 |
| 192 | 36.9984 | 45.5921 | 40.3871 | 50.7229 | 2.80376 |
| 191 | 31.9759 | 38.5069 | 28.4852 | 36.3372 | 1.37863 |
| 190 | 24.3131 | 30.3911 | 16.3151 | 21.2901 | -0.12467 |

**S6_raw_table (Fluorescence in presence of alcohol)**

| Wavelength | Native | a10 | a20 | a55 | a90 |
| --- | --- | --- | --- | --- | --- |
| 300 | 107.03 | 130.155 | 148.438 | 83.588 | 75.74 |
| 301 | 116.299 | 142.372 | 163.297 | 91.961 | 83.931 |
| 302 | 125.595 | 155.061 | 178.26 | 100.943 | 92.4 |
| 303 | 135.356 | 167.538 | 193.294 | 110.428 | 101.61 |
| 304 | 145.118 | 179.949 | 207.811 | 120.184 | 111.647 |
| 305 | 154.99 | 192.212 | 222.212 | 130.738 | 122.17 |
| 306 | 165.193 | 204.484 | 236.356 | 141.497 | 133.403 |
| 307 | 175.376 | 216.319 | 249.755 | 152.481 | 144.699 |
| 308 | 185.957 | 227.958 | 262.719 | 163.623 | 156.113 |
| 309 | 196.237 | 239.373 | 275.896 | 175.192 | 167.765 |
| 310 | 206.541 | 251.49 | 289.3 | 187.123 | 179.881 |
| 311 | 217.445 | 263.964 | 302.232 | 199.048 | 191.858 |
| 312 | 229.006 | 276.934 | 315.634 | 210.961 | 203.796 |
| 313 | 240.975 | 290.397 | 329.24 | 223.318 | 215.672 |
| 314 | 252.609 | 304.26 | 343.581 | 235.737 | 228.439 |
| 315 | 265.232 | 318.588 | 358.709 | 247.917 | 240.758 |
| 316 | 278.382 | 333.478 | 373.897 | 260.398 | 252.832 |
| 317 | 291.45 | 348.462 | 389.952 | 273.271 | 264.619 |
| 318 | 304.772 | 363.799 | 406.061 | 286.028 | 276.401 |
| 319 | 318.062 | 379.144 | 423.051 | 298.398 | 287.877 |
| 320 | 331.199 | 394.999 | 439.491 | 310.036 | 299.275 |
| 321 | 343.786 | 410.562 | 456.07 | 321.329 | 309.828 |
| 322 | 356.731 | 425.809 | 471.6 | 331.583 | 320.515 |
| 323 | 368.971 | 440.078 | 485.906 | 341.864 | 330.407 |
| 324 | 380.51 | 452.921 | 499.234 | 351.423 | 339.475 |
| 325 | 390.717 | 464.81 | 511.65 | 360.678 | 347.521 |
| 326 | 400.737 | 475.496 | 522.146 | 369.001 | 355.019 |
| 327 | 409.806 | 484.129 | 531.012 | 376.255 | 361.529 |
| 328 | 418.203 | 492.129 | 538.879 | 382.823 | 367.662 |
| 329 | 425.914 | 498.51 | 544.733 | 389.277 | 373.243 |
| 330 | 433.06 | 504.322 | 549.952 | 394.4 | 378.485 |
| 331 | 440.051 | 509.491 | 553.727 | 399.417 | 382.179 |
| 332 | 446.1 | 514.055 | 556.576 | 403.694 | 385.258 |
| 333 | 451.473 | 517.688 | 559.125 | 407.063 | 387.888 |
| 334 | 456.134 | 521.559 | 560.991 | 410.051 | 389.636 |
| 335 | 459.707 | 524.867 | 562.625 | 411.664 | 390.464 |
| 336 | 462.481 | 527.768 | 562.871 | 412.694 | 391.211 |
| 337 | 464.406 | 529.979 | 563.047 | 412.767 | 391.03 |
| 338 | 466.083 | 531.489 | 562.139 | 412.01 | 390.898 |
| 339 | 466.213 | 532.111 | 560.329 | 410.972 | 389.931 |
| 340 | 465.694 | 531.271 | 557.777 | 408.782 | 388.16 |
| 341 | 464.656 | 529.559 | 554.693 | 406.305 | 385.563 |
| 342 | 462.539 | 526.178 | 550.884 | 403.083 | 382.77 |
| 343 | 459.844 | 522.193 | 546.058 | 399.214 | 379.645 |
| 344 | 457.149 | 518.153 | 540.745 | 395.164 | 376.025 |
| 345 | 453.843 | 513.201 | 534.653 | 390.913 | 371.968 |
| 346 | 450.033 | 508.162 | 528.718 | 385.806 | 367.454 |
| 347 | 446.031 | 502.488 | 520.969 | 380.777 | 362.323 |
| 348 | 441.291 | 496.395 | 513.386 | 375.296 | 356.859 |
| 349 | 436.122 | 489.511 | 505.24 | 369.596 | 351.067 |
| 350 | 430.437 | 483.552 | 496.089 | 363.918 | 344.648 |
| 351 | 424.332 | 476.131 | 487.075 | 357.204 | 338.254 |
| 352 | 417.863 | 468.559 | 477.524 | 351.034 | 331.627 |
| 353 | 411.126 | 460.556 | 468.338 | 343.874 | 324.783 |
| 354 | 403.993 | 451.744 | 458.796 | 336.783 | 317.696 |
| 355 | 396.439 | 442.72 | 448.3 | 329.692 | 310.494 |
| 356 | 388.776 | 432.919 | 438.219 | 322.454 | 303.541 |
| 357 | 380.795 | 423.185 | 427.687 | 315.027 | 296.259 |
| 358 | 372.398 | 412.829 | 417.549 | 307.573 | 289.367 |
| 359 | 364.988 | 402.983 | 407.299 | 299.719 | 282.217 |
| 360 | 357.44 | 392.746 | 397.242 | 292.194 | 274.784 |
| 361 | 349.344 | 383.347 | 387.729 | 284.429 | 267.82 |
| 362 | 341.482 | 373.653 | 378.18 | 276.725 | 260.895 |
| 363 | 333.915 | 363.656 | 367.897 | 269.035 | 253.941 |
| 364 | 325.755 | 354.165 | 357.832 | 261.587 | 246.833 |
| 365 | 317.426 | 344.35 | 347.905 | 253.97 | 239.292 |
| 366 | 309.149 | 335.459 | 337.181 | 246.3 | 231.869 |
| 367 | 300.62 | 325.691 | 326.966 | 238.409 | 224.58 |
| 368 | 292.099 | 316.358 | 316.544 | 231.188 | 217.402 |
| 369 | 283.183 | 307.094 | 306.792 | 224.018 | 210.537 |
| 370 | 274.353 | 297.718 | 297.16 | 217.02 | 202.942 |
| 371 | 266.005 | 288.466 | 286.804 | 210.057 | 196.245 |
| 372 | 257.355 | 279.52 | 277.314 | 203.178 | 189.78 |
| 373 | 249.184 | 270.515 | 267.364 | 196.431 | 183.321 |
| 374 | 241.378 | 261.372 | 257.82 | 190.056 | 177.089 |
| 375 | 233.932 | 252.648 | 248.775 | 183.471 | 170.879 |
| 376 | 226.157 | 243.965 | 239.68 | 177.299 | 164.841 |
| 377 | 218.531 | 236.046 | 231.203 | 170.978 | 159.585 |
| 378 | 211.163 | 227.584 | 222.767 | 164.715 | 154.256 |
| 379 | 204.006 | 219.424 | 214.487 | 158.505 | 148.935 |
| 380 | 196.667 | 211.713 | 206.415 | 152.298 | 143.116 |
| 381 | 189.322 | 203.743 | 198.889 | 146.331 | 137.285 |
| 382 | 182.134 | 196.192 | 190.709 | 140.307 | 131.924 |
| 383 | 174.837 | 188.49 | 182.817 | 134.283 | 126.429 |
| 384 | 167.658 | 180.686 | 175.093 | 128.78 | 121.196 |
| 385 | 160.564 | 172.9 | 167.868 | 123.138 | 115.878 |
| 386 | 153.808 | 164.963 | 160.542 | 116.943 | 110.086 |
| 387 | 147.193 | 157.246 | 152.975 | 111.36 | 105.15 |
| 388 | 140.129 | 149.714 | 145.863 | 106.05 | 99.967 |
| 389 | 133.753 | 142.397 | 138.478 | 101.076 | 94.987 |
| 390 | 127.207 | 135.307 | 131.357 | 96.246 | 90.353 |
| 391 | 120.861 | 128.465 | 124.818 | 91.443 | 85.94 |
| 392 | 114.52 | 122.058 | 118.22 | 86.871 | 81.626 |
| 393 | 108.933 | 115.893 | 112.033 | 82.426 | 77.572 |
| 394 | 103.723 | 109.976 | 106.366 | 78.211 | 73.658 |
| 395 | 98.921 | 104.677 | 100.854 | 74.18 | 69.76 |
| 396 | 94.262 | 99.723 | 95.931 | 70.573 | 66.087 |
| 397 | 90.034 | 95.104 | 91.343 | 67.189 | 62.778 |
| 398 | 86.125 | 90.97 | 87.012 | 63.983 | 59.791 |
| 399 | 82.228 | 86.929 | 83.113 | 60.944 | 57.012 |
| 400 | 78.876 | 83.09 | 79.491 | 58.208 | 54.557 |
| 401 | 75.482 | 79.652 | 76.297 | 55.441 | 52.166 |
| 402 | 72.262 | 76.357 | 73.098 | 52.812 | 49.891 |
| 403 | 69.288 | 73.581 | 70.318 | 50.774 | 47.937 |
| 404 | 66.437 | 70.788 | 67.831 | 48.721 | 46.014 |
| 405 | 64.131 | 68.432 | 65.392 | 46.835 | 44.197 |
| 406 | 61.923 | 66.13 | 63.473 | 45.072 | 42.532 |
| 407 | 59.816 | 64.085 | 61.545 | 43.348 | 41.045 |
| 408 | 57.887 | 62.066 | 59.617 | 41.899 | 39.455 |
| 409 | 56.224 | 60.262 | 57.826 | 40.588 | 37.95 |
| 410 | 54.586 | 58.365 | 56.072 | 39.224 | 36.647 |
| 411 | 52.89 | 56.649 | 54.57 | 37.922 | 35.305 |
| 412 | 51.278 | 55.001 | 53.123 | 36.764 | 34.179 |
| 413 | 49.657 | 53.411 | 51.633 | 35.611 | 33.069 |
| 414 | 48.03 | 51.861 | 50.305 | 34.531 | 32.085 |
| 415 | 46.472 | 50.2 | 48.865 | 33.435 | 31.293 |
| 416 | 45.201 | 48.679 | 47.348 | 32.401 | 30.336 |
| 417 | 43.636 | 47.263 | 45.82 | 31.369 | 29.295 |
| 418 | 42.289 | 45.642 | 44.258 | 30.327 | 28.288 |
| 419 | 40.939 | 44.326 | 42.959 | 29.381 | 27.41 |
| 420 | 39.683 | 42.814 | 41.721 | 28.41 | 26.427 |
| 421 | 38.469 | 41.392 | 40.512 | 27.467 | 25.41 |
| 422 | 37.212 | 40.157 | 39.476 | 26.56 | 24.452 |
| 423 | 35.946 | 38.801 | 38.271 | 25.51 | 23.573 |
| 424 | 34.92 | 37.823 | 37.282 | 24.765 | 22.573 |
| 425 | 33.947 | 36.862 | 36.282 | 23.893 | 21.792 |
| 426 | 32.974 | 35.839 | 35.426 | 23.037 | 21.04 |
| 427 | 31.854 | 34.967 | 34.73 | 22.397 | 20.458 |
| 428 | 30.938 | 34.004 | 33.906 | 21.747 | 19.805 |
| 429 | 30.02 | 33.288 | 33.153 | 21.214 | 19.359 |
| 430 | 29.283 | 32.553 | 32.526 | 20.583 | 18.877 |
| 431 | 28.558 | 31.781 | 31.762 | 20.09 | 18.437 |
| 432 | 27.866 | 31.124 | 31.173 | 19.543 | 17.9 |
| 433 | 27.054 | 30.518 | 30.453 | 18.972 | 17.327 |
| 434 | 26.42 | 29.758 | 29.828 | 18.388 | 16.919 |
| 435 | 25.698 | 29.19 | 29.186 | 17.978 | 16.565 |
| 436 | 24.971 | 28.523 | 28.502 | 17.458 | 16.133 |
| 437 | 24.281 | 27.881 | 27.814 | 16.935 | 15.75 |
| 438 | 23.598 | 27.262 | 27.04 | 16.358 | 15.326 |
| 439 | 23.117 | 26.577 | 26.428 | 15.882 | 14.888 |
| 440 | 22.767 | 26.096 | 25.844 | 15.513 | 14.479 |
| 441 | 22.352 | 25.39 | 25.271 | 15.162 | 14.221 |
| 442 | 21.755 | 24.829 | 24.725 | 14.844 | 13.806 |
| 443 | 21.755 | 24.829 | 24.725 | 14.844 | 13.806 |
| 444 | 21.755 | 24.829 | 24.725 | 14.844 | 13.806 |
| 445 | 21.755 | 24.829 | 24.725 | 14.844 | 13.806 |
| 446 | 21.755 | 24.829 | 24.725 | 14.844 | 13.806 |
| 447 | 21.755 | 24.829 | 24.725 | 14.844 | 13.806 |
| 448 | 21.755 | 24.829 | 24.725 | 14.844 | 13.806 |
| 449 | 21.755 | 24.829 | 24.725 | 14.844 | 13.806 |
| 450 | 21.755 | 24.829 | 24.725 | 14.844 | 13.806 |

**S7_raw_table (CD in presence of salt)**

| Wavelength | native | S50 | S100 | S300 |
| --- | --- | --- | --- | --- |
| 250 | 0.34323 | 0.3816 | 0.38218 | 0.46934 |
| 249 | 0.32116 | 0.33745 | 0.29826 | 0.41504 |
| 248 | 0.23284 | 0.21571 | 0.16098 | 0.26531 |
| 247 | 0.1079 | 0.02237 | 0.00183 | 0.02752 |
| 246 | -0.02567 | -0.18772 | -0.17739 | -0.23088 |
| 245 | -0.19364 | -0.38698 | -0.40226 | -0.47596 |
| 244 | -0.40235 | -0.65798 | -0.69009 | -0.80927 |
| 243 | -0.69667 | -1.03514 | -1.0681 | -1.27316 |
| 242 | -1.09628 | -1.41913 | -1.47995 | -1.74544 |
| 241 | -1.50593 | -1.78544 | -1.87187 | -2.19598 |
| 240 | -2.00481 | -2.31882 | -2.48718 | -2.852 |
| 239 | -2.59211 | -3.0558 | -3.263 | -3.75844 |
| 238 | -3.34135 | -3.94944 | -4.14208 | -4.85757 |
| 237 | -4.24585 | -4.95391 | -5.23267 | -6.093 |
| 236 | -5.28497 | -6.14388 | -6.53794 | -7.55659 |
| 235 | -6.50372 | -7.46208 | -7.98194 | -9.1779 |
| 234 | -7.83906 | -8.88421 | -9.61411 | -10.92703 |
| 233 | -9.32563 | -10.486 | -11.4281 | -12.89713 |
| 232 | -11.0246 | -12.2859 | -13.3504 | -15.1109 |
| 231 | -12.8006 | -14.1941 | -15.3958 | -17.45786 |
| 230 | -14.6411 | -16.0878 | -17.4776 | -19.787 |
| 229 | -16.3863 | -18.0068 | -19.4106 | -22.14725 |
| 228 | -18.0587 | -19.7654 | -21.4118 | -24.31022 |
| 227 | -19.6746 | -21.3513 | -23.2564 | -26.26078 |
| 226 | -21.1643 | -22.741 | -24.845 | -27.97002 |
| 225 | -22.2577 | -23.8068 | -26.17 | -29.28089 |
| 224 | -23.0651 | -24.5634 | -27.1799 | -30.21146 |
| 223 | -23.6442 | -25.154 | -27.8782 | -30.93786 |
| 222 | -23.9336 | -25.6197 | -28.3832 | -31.51064 |
| 221 | -24.0913 | -25.9519 | -28.5457 | -31.91923 |
| 220 | -24.1369 | -26.1215 | -28.44 | -32.12783 |
| 219 | -23.9167 | -26.1247 | -28.2011 | -32.13176 |
| 218 | -23.7064 | -26.0439 | -27.9719 | -32.03238 |
| 217 | -23.4918 | -25.9706 | -27.7434 | -31.94223 |
| 216 | -23.2458 | -25.6549 | -27.5474 | -31.55394 |
| 215 | -23.0196 | -25.438 | -27.3704 | -31.28716 |
| 214 | -23.1567 | -25.5682 | -27.4968 | -31.4473 |
| 213 | -23.5559 | -25.8448 | -27.9434 | -31.7875 |
| 212 | -24.1496 | -26.5225 | -28.7689 | -32.62103 |
| 211 | -25.0641 | -27.7752 | -30.0512 | -34.16177 |
| 210 | -26.2589 | -29.2829 | -31.526 | -36.01615 |
| 209 | -27.6264 | -30.831 | -32.8257 | -37.92022 |
| 208 | -28.8995 | -32.0154 | -33.7566 | -39.37696 |
| 207 | -29.8329 | -32.9082 | -34.1535 | -40.47505 |
| 206 | -30.3064 | -33.1734 | -33.8056 | -40.80123 |
| 205 | -29.8293 | -32.8803 | -32.5552 | -40.44073 |
| 204 | -28.1721 | -31.7054 | -30.1763 | -38.99568 |
| 203 | -24.9661 | -28.9103 | -27.3498 | -35.55788 |
| 202 | -21.5064 | -24.7153 | -22.5748 | -30.39829 |
| 201 | -17.2903 | -19.6979 | -15.0742 | -24.2272 |
| 200 | -11.5426 | -14.3634 | -7.78323 | -17.66609 |
| 199 | -5.54758 | -6.21806 | 0.765 | -7.64783 |
| 198 | 2.36437 | 4.83502 | 13.5186 | 5.94677 |
| 197 | 14.9626 | 16.9776 | 24.167 | 20.8814 |
| 196 | 26.9969 | 26.0178 | 30.578 | 32.00028 |
| 195 | 34.9234 | 31.2337 | 33.4575 | 38.41551 |
| 194 | 38.946 | 32.9496 | 33.0512 | 40.52597 |
| 193 | 39.5116 | 31.2598 | 29.6332 | 38.44762 |
| 192 | 36.9984 | 27.0369 | 23.8559 | 33.25371 |
| 191 | 31.9759 | 21.466 | 16.6282 | 26.40185 |
| 190 | 24.3131 | 14.6556 | 10.49 | 18.02548 |

**Table S7_raw_data (Acrylamide quenching)**

|  | pH-2 | pH-3 | pH-5 | pH-7 | pH-10 |
| --- | --- | --- | --- | --- | --- |
| Best-fit value | | | | | |
| Slope | 21.28 ± 0.5306 | 19.26 ± 0.5108 | 9.019 ± 0.5265 | 6.469 ± 0.8686 | 7.177 ± 0.3751 |
| Y-intercept when X=0.0 | 1.076 ± 0.07567 | 1.122 ± 0.07284 | 0.9979 ± 0.07507 | 0.9779 ± 0.1239 | 0.8763 ± 0.05349 |
| X-intercept when Y=0.0 | -0.05056 | -0.05824 | -0.1106 | -0.1512 | -0.1221 |
| 1/slope | 0.04699 | 0.05191 | 0.1109 | 0.1546 | 0.1393 |
| 95% Confidence Intervals | | | | | |
| Slope | 20.10 to 22.46 | 18.12 to 20.40 | 7.846 to 10.19 | 4.534 to 8.404 | 6.341 to 8.012 |
| Y-intercept when X=0.0 | 0.9073 to 1.245 | 0.9596 to 1.284 | 0.8307 to 1.165 | 0.7019 to 1.254 | 0.7571 to 0.9955 |
| X-intercept when Y=0.0 | -0.06145 to -0.04070 | -0.07030 to -0.04740 | -0.1465 to -0.08262 | -0.2692 to -0.08581 | -0.1552 to -0.09560 |
| Goodness of Fit | | | | | |
| r² | 0.9938 | 0.9930 | 0.9670 | 0.8473 | 0.9734 |
| Sy.x | 0.1507 | 0.1451 | 0.1495 | 0.2467 | 0.1065 |
| Is slope significantly non-zero? | | | | | |
| F | 1608 | 1422 | 293.5 | 55.47 | 366.0 |
| DFn, DFd | 1.000, 10.00 | 1.000, 10.00 | 1.000, 10.00 | 1.000, 10.00 | 1.000, 10.00 |
| P value | < 0.0001 | < 0.0001 | < 0.0001 | < 0.0001 | < 0.0001 |
| Deviation from zero? | Significant | Significant | Significant | Significant | Significant |
| Data | | | | | |
| Number of X values | 12 | 12 | 12 | 12 | 12 |
| Maximum number of Y replicates | 1 | 1 | 1 | 1 | 1 |
| Total number of values | 12 | 12 | 12 | 12 | 12 |
| Number of missing values | 0 | 0 | 0 | 0 | 0 |
