# Supplementary material for: An insight into structural plasticity and conformational transitions of transcriptional co-activator Sus1
Source: PLoS One. 2020 Mar 5;15(3):e0229216. doi: 10.1371/journal.pone.0229216 (PMC7058303; doi:10.1371/journal.pone.0229216)
Supplement: S1 Fig — Mean residue ellipticity value (MRE) of the far UV-CD of Sus1 at 222 nm and 208 nm was plotted at different pH. MRE value at 222 nm and 208 nm is represented by filled square (■) and open circle (○) respectively. (DOCX) [file pone.0229216.s002.docx]

**Fig-S1**
