# Supplementary material for: An insight into structural plasticity and conformational transitions of transcriptional co-activator Sus1
Source: PLoS One. 2020 Mar 5;15(3):e0229216. doi: 10.1371/journal.pone.0229216 (PMC7058303; doi:10.1371/journal.pone.0229216)
Supplement: S2 Fig — AKTA profile of Sus1 at different pH. AKTA profile at pH7, pH5 and pH2 is represented by the closed circle (●), closed tringle (▲) and open circle (○) respectively. (DOCX) [file pone.0229216.s003.docx]

**Fig-S2**
